# SET1B Drives Sustained HIF Activity and Disease Progression in Clear-Cell Renal Cell Carcinoma

**DOI:** 10.1158/0008-5472.CAN-25-1076

**Published:** 2026-04-06

**Authors:** Brian M. Ortmann, Tekle Pauzaite, James A.C. Bertlin, Rachel V. Seear, Esther Arnaiz, Lukasz W. Marzec, Alexander Handyside, Laura M. Bowker, Sarah E. Barnett, Katherine Harcourt, Salwa Lin, Ahmed M. Salman, Laura Wilson, Adrian L. Harris, Grant D. Stewart, Anastasia Hepburn, Simon Clare, Craig N. Robson, Judy M. Coulson, Anneliese O. Speak, James A. Nathan

**Affiliations:** 1 Cambridge Institute of Therapeutic Immunology and Infectious Disease (CITIID), Jeffrey Cheah Biomedical Centre, Department of Medicine, University of Cambridge, Cambridge, United Kingdom.; 2 Biosciences Institute, Paul O’Gorman Building, Newcastle University, Newcastle, United Kingdom.; 3 Egg Facility, Liverpool Shared Research Facilities, Faculty of Health and Life Sciences, University of Liverpool, Liverpool, United Kingdom.; 4 Cancer Research UK Department of Medical Oncology, Weatherall Institute of Molecular Medicine, University of Oxford, Oxford, United Kingdom.; 5 The Jenner Institute, NDM, University of Oxford, Oxford, United Kingdom.; 6 Translational and Clinical Research Institute, Paul O’Gorman Building, Newcastle University, Newcastle, United Kingdom.; 7 Department of Surgery, University of Cambridge, Cambridge, United Kingdom.; 8 Cancer Research UK Cambridge Centre, Cambridge Biomedical Campus, Cambridge, United Kingdom.; 9 Molecular and Clinical Cancer Medicine, Institute of Systems, Molecular and Integrative Biology, University of Liverpool, Liverpool, United Kingdom.

## Abstract

The cellular response to hypoxia is driven by hypoxia-inducible factors (HIF), which regulate genes involved in glycolysis, angiogenesis, and cell proliferation, as well as inflammation and tumor progression. HIF activation is well characterized and is primarily regulated by oxygen-dependent prolyl hydroxylation and subsequent degradation. SET1B, a histone H3 lysine 4 methyltransferase, has recently emerged as a key modulator of HIF target gene transcription, but evidence suggests that it plays a broader role in modulating HIF transcriptional activity beyond histone methylation. In this study, we revealed that SET1B interacts with RNA polymerase II to coordinate sustained HIF-mediated transcriptional activity through multiple functional domains. In clear-cell renal cell carcinoma (ccRCC), SET1B is critical for sustained HIF activity, and SET1B expression correlated with disease progression and metastasis in patient samples. Moreover, SET1B depletion enhanced the efficacy of HIF2 inhibitors. These findings establish SET1B as a driver of tumor progression and potential therapeutic target in ccRCC.

**Significance:**

SET1B functions as a key regulator of HIF-dependent transcription and cancer growth under low-oxygen conditions, revealing a therapeutic target to enhance treatment efficacy and potentially slow disease progression in kidney cancer.

## Introduction

Cellular responses to hypoxia are essential for the survival of multicellular organisms and involve transcriptional reprogramming driven by the hypoxia-inducible factor (HIF) family of transcription factors (1). HIFs regulate genes critical for glycolysis, angiogenesis, and cell proliferation (2). Moreover, HIFs also play roles in inflammation, immune modulation, and tumor progression, making the HIF pathway a promising therapeutic target (3–5).

HIF regulation primarily occurs posttranscriptionally via oxygen-dependent degradation of the two main HIFα isoforms, HIF1α and HIF2α. Under normoxic conditions, HIF undergoes oxygen-dependent prolyl hydroxylation by prolyl hydroxylase domain (PHD) enzymes, facilitating the binding of the von Hippel–Lindau (VHL) E3 ubiquitin ligase complex, leading to ubiquitination and proteasomal degradation (6–10). Under hypoxic conditions, decreased PHD activity stabilizes HIFα, promoting dimerization with HIF1β to form a heterodimer complex that activates gene transcription.

Although the mechanisms underlying HIF activation are well characterized, how HIFs transcriptionally regulate individual target genes across diverse cellular and pathologic contexts remains an important area of investigation. This has significant clinical implications, as targeting HIF presents an attractive therapeutic strategy. However, the potential toxicity associated with broadly suppressing HIF activity remains a challenge. Transcriptional coactivators and epigenetic modifications have been shown to facilitate HIF specificity, offering an alternative approach to target specific pathways of HIF activity (11–13). Early studies identified CBP/p300 as coactivators for subsets of HIF target genes (14), whereas more recent research has uncovered roles for other coactivators, including TIP60, CDK8 mediator, DNA-PK, and the FACT complex (15–18). HIF must also interact with the transcriptional machinery, as transcriptional outcomes are largely dependent on RNA polymerase II (Pol II) activity (19, 20). An accepted role of transcriptional activators is to promote Pol II recruitment and initiation. However, recent evidence suggests that Pol II often initiates transcription but pauses shortly downstream of the transcriptional start site, a phenomenon observed in 40% to 70% of genes involved in stress responses, including hypoxia (21–23). Therefore, how HIF and associated transcriptional activators interact with Pol II is not well understood.

We recently discovered that the SET1B histone 3 lysine 4 (H3K4) methyltransferase plays a crucial role in regulating the transcription of HIF target genes, particularly those typically expressed at low levels under normoxic conditions (24). Although this effect of SET1B could be attributed to increased histone methylation during hypoxia, given that H3K4 methylation is traditionally associated with active transcription (25), H3K4 trimethylation (H3K4me3) levels did not consistently correlate with reduced mRNA expression. In fact, depletion of SET1B led to a reduction in H3K4me3 levels across gene bodies rather than being limited to transcriptional start sites of specific HIF target genes, indicating that SET1B plays a broader role in modulating HIF transcriptional activity. SET1B also contains additional functional domains beyond its known SET enzymatic domain, but how these regions contribute to HIF-mediated transcription is not known.

Here, we demonstrate that multiple functional domains of SET1B, including its interaction with RNA Pol II, are critical for modulating HIF transcriptional activity under hypoxic conditions. Using a model of sustained HIF activation, we also show that SET1B is essential not only for initiating HIF activity but for maintaining its prolonged activation. These findings have direct relevance to human disease. Using clear-cell renal cell carcinoma (ccRCC) as cancer type in which sustained HIF activity is central to tumorigenesis, we find that SET1B is indispensable for maintaining HIF activity. Importantly, SET1B expression also correlates strongly with disease progression in patient samples. Furthermore, SET1B depletion enhances the efficacy of a clinically relevant HIF2 inhibitor and reduces HIF-dependent transcription, even in cases of resistance to HIF2 inhibitors. These findings establish SET1B as a driver of HIF-dependent ccRCC progression and emphasize its potential as a therapeutic target in ccRCC.

## Materials and Methods

### Cell lines and reagents

The 786-0 wild-type (WT) and 769-P cells were maintained in RPMI-1640 (Sigma-Aldrich) supplemented with 10% fetal calf serum (FCS). HeLa, A549, HEK293T, RCC4, and RCC10 cells were maintained in DMEM (Sigma-Aldrich) supplemented with 10% FCS. Cells were maintained in a 5% CO_2_ incubator at 37°C. All cell lines were purchased from ATCC. PT2385 was acquired from BioVision (B1920) and was added to cell cultures at indicated concentrations. Cells were confirmed *Mycoplasma* negative (Lonza, MycoAltert) and authenticated by short tandem repeat profiling (Eurofins Genomics).

### Plasmids

Plasmids used were pKLV-U6gRNA-EF(BbsI)-PGKpuro2ABFP (Addgene plasmid, cat. #62348), lentiCRISPRv2 [single-guide RNA (sgRNA)/Cas9, F. Zhang Addgene cat. #52961], pHRSIN-pSFFV-HA-pPGK-Puro, pHRSIN-pSFFV-pPGK-Puro, pMD.G (lentiviral VSVG), and pMD.GagPol (lentiviral gag/pol). HIF2 truncation mutant constructs were generated from pCDNA3 HIF2α and cloned into the pHRSIN-pSFFV backbone with puromycin resistance using NEBuilder HiFi (NEB). pCDNA3 HIF2 was cloned into the pHRSIN-pSFFV backbone, and Gibson cloning was used to generate the G323E mutant. Primers for cloning the G323E and truncation mutants are shown in Supplementary Table S1. The SET1B construct was a gift from D. Skalnik’s laboratory. SET1B was cloned into the pHRSIN-pSFFV backbone with puromycin resistance using NEBuilder HiFi (NEB) as described previously (24). Primers for HIF2 cloning are shown in Supplementary Table S1.

### Lentiviral production and transduction

Lentivirus was produced by transfection of HEK293T cells as previously described (24) using FuGENE (Promega) at 70% to 80% confluency in 6-well plates, with the appropriate pHRSIN vector and the packaging vectors pCMVR8.91 (gag/pol) and pMD.G (VSVG). The viral supernatant was harvested at 48  hours, filtered (0.45-μm filter), and stored at −80°C. For transduction, cells were seeded on 24-well plates in 500  µL medium, 500  µL viral supernatant was added, and plates were centrifuged at 1,800  rpm., 37°C for 1 hour. Antibiotic selection was applied from 48  hours.

### CRISPR–Cas9 targeted deletions

As described previously, gene-specific CRISPR sgRNA sequences were taken from the TKO library or designed using E-CRISP (http://www.e-crisp.org/E-CRISP/), with 5′-CACC and 3′-CAAA overhangs, respectively (24). sgRNAs were ligated into the lentiCRISPRv2 or pKLV-U6gRNA(BbsI)-PGKpuro2ABFP vector and lentivirus produced as described. Transduced cells were selected with puromycin and were generally cultured for 9 to 10 days before subsequent experiments to allow sufficient times for depletion of the target protein. Knockout (KO) clones were isolated from the sgRNA-targeted populations by serial dilution or FACS. sgRNAs used are shown in Supplementary Table S2.

### Immunoblotting

As described previously, cells were lysed in an SDS lysis buffer [1% SDS, 50  mmol/L Tris (pH 7.4), 150  mmol/L NaCl, 10% glycerol, and 5  μL mL^−1^ Benzonase (Sigma-Aldrich)] for 10  minutes before heating at 90°C for 5 minutes (24). Proteins were separated by SDS-PAGE, transferred to polyvinylidene difluoride membranes, probed with appropriate primary and secondary antibodies, and developed using enhanced chemiluminescent or SuperSignal West Pico PLUS Chemiluminescent substrate (Thermo Fisher Scientific). All antibodies and reagents are listed in Supplementary Table S3.

### Quantitative PCR

Total RNA was extracted using the RNeasy Plus minikit (Qiagen) following the manufacturer’s instructions and then reversed-transcribed using ProtoScript II Reverse Transcriptase (NEB) as previously described (24). Template cDNA (20 ng) was amplified using the ABI 7900HT Real-Time PCR system (Applied Biotechnology or QuantStudio 7, Thermo Fisher Scientific). Transcript levels of genes were normalized to a reference index of a housekeeping gene (*β*-*actin*). Primer sequences are shown in Supplementary Table S4.

### Immunoprecipitation

As previously described, 786-0 cells were lysed in 1% Triton TBS, with 1× Roche complete EDTA-free protease inhibitor cocktail for 30  minutes at 4°C (24). Lysates were centrifuged at 14,000  rpm. for 10  minutes, supernatants collected, and then diluted to 0.1% detergent for preclearing with Protein G magnetic beads (Thermo Fisher Scientific) for 2  hours at 4°C. Supernatants were then incubated with a primary antibody overnight (rotation at 4°C). Protein G magnetic beads were then added for 2  hours, and samples were then washed 3 times. Bound proteins were eluted in 2× SDS loading buffer, separated by SDS-PAGE, and immunoblotted.

### Subcellular fractionation

As previously described, a total of 10  ×  10^6^ 786-0 cells were washed in PBS, lysed in buffer A (10  mmol/L HEPES, 1.5  mmol/L MgCl_2_, 10  mmol/L KCl, 0.5  mmol/L dithiothreitol (DTT), 0.1% NP40 and ethylenediaminetetraacetic acid (EDTA)-free protease inhibitor cocktail tablet, Roche), and incubated with rotation at 4°C for 10  minutes (24). The supernatants containing cytosolic fractions were collected by centrifugation (1,400 × *g* for 4  minutes at 4°C). The nuclear pellet was resuspended in buffer B (20  mmol/L HEPES, 1.5  mmol/L MgCl_2_, 300  mmol/L NaCl, 0.5  mmol/L DTT, 25% glycerol, 0.2  mmol/L EDTA, and EDTA-free protease inhibitor cocktail tablet) for 10  minutes on ice to separate nucleoplasmic and chromatin fractions. Samples were centrifuged at 1,700 × *g* for 4  minutes at 4°C, separating the soluble nucleoplasm from the insoluble chromatin fraction. The chromatin fraction was solubilized in 2× SDS loading buffer containing 1:500 Benzonase (Sigma-Aldrich).

### VEGF ELISA

As previously described, cells were plated in 6-well plates and treated with 1% or 21% O_2_ for 24  hours (24). Culture supernatants were collected, centrifuged at 1,500  rpm for 10  minutes at 4°C, and analyzed using the Human VEGF Quantikine ELISA kit (R&D Systems) according to the manufacturer’s instructions.

### Chromatin immunoprecipitation–quantitative PCR

As previously described, 786-0 and HeLa cells were grown on 15-cm dishes up to a 2  ×  10^6^ density and treated with 1% formaldehyde for 10  minutes to cross-link proteins to chromatin (24). The reaction was quenched with glycine (0.125  mol/L for 10  minutes at room temperature). Cells were then washed in ice-cold PBS twice, scraped in tubes, and centrifuged at 800  rpm for 10  minutes before lysis in 500  µL of chromatin immunoprecipitation (ChIP) lysis buffer [50  mmol/L Tris-HCl (pH 8.1), 1% SDS, 10 mmol/L EDTA, and cOmplete Mini EDTA-free protease inhibitor]. Samples were incubated on ice for 10  minutes and diluted 1:1 with ChIP dilution buffer (20  mmol/L Tris-HCl (pH 8.1), 1% (v/v) Triton X-100, 2  mmol/L EDTA, and 150  mmol/L NaCl). Samples were then sonicated in tubes and beads for 20 cycles of 30  seconds on and 30 seconds off in a Bioruptor (Diagenode), followed by centrifugation for 10  minutes at 13,000  rpm. at 4°C. Supernatants were collected and 20  µL stored at −20°C as the input sample. A 200  µL aliquot of the remaining sample was diluted with ChIP dilution buffer to 1 mL and precleared using 20  µL Protein G magnetic beads (4°C, 2  hours, rotating); 1 mL of sample was immunoprecipitated with the appropriate primary antibody (4°C, overnight, rotating). Protein G magnetic beads (25 μL) were added and incubated for an additional 2  hours at 4°C. The beads were washed sequentially for 5  minutes each with wash buffer 1 [20  mmol/L Tris-HCl (pH 8.1), 0.1% (w/v) SDS, 1% (v/v) Triton X-100, 2  mmol/L EDTA, and 150  mmol/L NaCl], wash buffer 2 (wash buffer 1 with 500  mmol/L NaCl), wash buffer 3 [10  mmol/L Tris-HCl (pH 8.1), 0.25  mol/L LiCl, 7 1% (v/v) NP-40, 1% (w/v) Na-deoxycholate, and 1  mmol/L EDTA], and twice with TE buffer [10  mmol/L Tris-HCl (pH 8.0) and 1  mmol/L EDTA]. Bound complexes were eluted with 120  μL elution buffer (1% (w/v) SDS and 0.1  mol/L Na-bicarbonate), and cross-linking was reversed by the addition of 0.2  mol/L NaCl, followed by incubation at 65°C overnight with agitation (300  rpm.). Protein was digested with 20  μg proteinase K (Thermo Fisher Scientific) for 4  hours at 45°C. RNase H (Thermo Fisher Scientific) was added for 30  minutes at 37°C and DNA purified using the DNA MinElute kit (Qiagen). DNA underwent qPCR analysis, and results were expressed relative to input material. Primer sequences are shown in Supplementary Table S4.

### Short hairpin RNA–mediated RNA depletion

Oligonucleotides were designed with a TTCAAGAGA hairpin, using short hairpin RNA (shRNA) sequences from the Broad Institute RNAi Consortium shRNA Library (Supplementary Table S2). Sequences were cloned into pC.SIREN.Puro by digestion and ligation. Cells were transduced with the indicated vector or a scrambled shRNA control, and assays performed after at least 7 days, following puromycin selection. The shRNA constructs were purchased from Sigma-Aldrich and cloned into the pSIREN vector. The clone IDs for SET1B are TRCN0000237963 (sh*SET1B*-#1) and TRCN0000237965 (shSET1B-#2). shRNA sequences are listed in Supplementary Table S2.

### siRNA-mediated depletion

Cells were transfected with siRNA SMARTpools for *SETD1B* (Dharmacon), SETD1B untranslated region (UTR) siRNA (Dharmacon), and Universal Negative Control using Lipofectamine RNAiMAX (Thermo Fisher Scientific). Cells were harvested after 48 hours for further analysis by flow cytometry, quantitative PCR (qPCR), or immunoblot.

### Analysis of The Cancer Genome Atlas expression and survival data

*SETD1B* mRNA expression and survival data for ccRCC tumors were obtained from The Cancer Genome Atlas (TCGA). The R package survminer was used to perform a log-rank test and plot a Kaplan–Meier curve.

### Migration assay

A total of 1.2 × 10^4^ 786-0 cells were seeded per well in 96-well ImageLock plates (4379, Essen BioScience) and incubated for 24 hours. A minimum of 18 wells per condition were seeded in each biological replicate. If the effect of PT2385 was tested, the compound was added to the wells once the cells were attached. The WoundMaker (4563, Essen BioScience) is a 96-spin mechanical device designed to create 700- to 800-μm-wide homogeneous wounds in cell monolayers on 96-well ImageLock plates. Prior to use after storage, the WoundMaker lid was washed with sterile distilled water for 5 minutes and with 70% ethanol for another 5 minutes. After washing, the scratch was performed to create precise wounds in all wells of the ImageLock plate. After wounding, cells were washed with fresh media to prevent dislodged cells from settling and reattaching. When PT2385 was tested, the new media contained 1 µmol/L PT2385. Once the wound was made, the plates were placed into the IncuCyte ZOOM for 24 hours in the case of 786-0 cells or 72 hours in the case of RCC4 cells. Scanning was performed using a 10× objective and scheduled every 2 hours. Migration ability of different cells and conditions was analyzed through two integrated metrics that IncuCyte software calculates based on the processed images: wound width and wound confluence. Wound width represents the average distance (μm) between the leading edge of the population of migrating cells (scratch wound mask) within an image. Wound confluence determines the percentage of the wound area that is occupied by cells, and it relies on the initial scratch wound mask to differentiate the wounded from the nonwounded region. Debris or other matter within the wound is quantified as confluence and is not background subtracted. Therefore, the wound confluence values at the initial time point may not be 0%.

### Chorioallantoic membrane assay

Fertilized chicken eggs (Medeggs Ltd.) were incubated and prepared as previously described (26) with 2 × 10^6^ cells implanted per egg on embryonic day 8 (E8). To ensure unbiased allocation of eggs to the treatment group, the three cell lines were first assigned to the treatment group A, B, or C by a second researcher. Each egg was given an ID number, assigned a random number using the Microsoft Excel function = RAND(), ranked using = RANK(), and then evenly split into three groups, with the first third allocated to group A, second third to group B, and final third to group C. On E14, the chorioallantoic membrane (CAM) of viable engrafted eggs was fixed for 5 minutes with 10% neutral buffered formalin (Sigma-Aldrich) *in situ*. Xenografts were imaged after dissection using a Zeiss SterREO Discovery.V12 microscope fitted with an Achromat S 1.0× objective and Axiocam 305 color camera. CAM vasculature of the dissected samples was analyzed using IKOSA CAM Assay software (V3.2.0, Kolaido GmbH) by a third researcher, blinded to the research hypothesis. Microvessel density (total vessel length/area analyzed) and relative branching points (branching points/area analyzed) were calculated. All experiments utilizing fertilized chicken eggs were terminated by E14 (two thirds of the gestation period), meaning that the model is classified as nonprotected under The Animals (Scientific Procedures) Act 1986 (amended 2012) in the United Kingdom and therefore home office approval was not required. Standard operating procedures were reviewed by the Liverpool Animal Welfare and Ethical Review Body.

#### Clinical samples

All patient tissue samples were used in accordance with ethical approval granted by the Northumberland, Tyne and Wear NHS Strategic Health Authority Research Ethics Committee (reference 2003/11; The Freeman Hospital), and written informed consent was obtained from all patients.

#### Immunohistochemistry analysis

Immunohistochemistry (IHC) was performed using tissue microarrays containing 0.6-mm cores of renal cancer and control kidney tissues as described (27). Sections were immunostained with anti-SET1B 1:100 (Atlas Antibodies HPA021667) and viewed using Aperio CS2 (Leica Biosystems). SET1B intensity was compared across normal versus tumor tissue and across the different clinical stages of ccRCC which were graded by two independent clinicians who were blinded, and the H score was calculated.

#### Cell invasion assay

ImageLock 96-well plates were coated with a thin layer of Matrigel Growth Factor Reduced Basement Membrane Matrix (354230, Corning). To this end, Matrigel stock solution was dissolved in cold cell culture media to a final concentration of 100 μg/mL. Wells were coated with 50 μL of the solution, and the plate was placed in an incubator at 37°C and 5% CO_2_ overnight. Matrigel was removed, and either 1.2 × 10^4^ cells in the case of the 786-0 cell line or 2 × 10^4^ in the case of the RCC4 cell line were seeded per well and incubated for 24 hours. In this case, a minimum of 10 wells per condition were used per biological replicate. When necessary, PT2385 was added to the wells once the cells were attached. Afterward, the scratch was performed to create precise wounds in all wells of the ImageLock plate. Wells were washed once with cell culture media, and 50 μL Matrigel (8 mg/mL) was added to each well carefully, avoiding the formation of bubbles. The plate was placed in the incubator for 30 minutes prior to the addition of 100 μL cell culture media containing PT2385 or not. The plate was then placed into IncuCyte ZOOM for 5 days. Scanning was performed using a 10× objective and scheduled every 4 hours. The WoundMaker was washed before and after using as described in “Migration assay.” The invasion ability was analyzed using the relative wound density (RWD). Like wound confluence, RWD also relies on the initial scratch wound mask to differentiate between cell-occupied and cell-free regions of the image because it calculates the density of the wound region relative to the density of the cell region. This parameter is the only recommended metric for cell invasion because (i) the mild texture of the extracellular matrix (ECM) can hinder the ability of the analysis algorithm to apply an appropriate initial scratch wound mask and confluence mask and consequently result in a misleading wound confluence metric and (ii) cells invading through a 3D matrix usually exhibit an elongated phenotype with filopodia that extend into the ECM. Furthermore, one leader cell is generally followed by numerous other cells, forming tracts, as opposed to a leading edge of cells seen in a migration assay. For this reason, the scratch wound masks may not best represent the invading population, and wound width measure can thus be inaccurate and misleading.

#### Luciferase mouse metastasis and imaging

Cells were harvested and washed in Dulbecco’s phosphate-buffered saline (D-PBS, without calcium and magnesium). Immunodeficient mice (NOD.Cg-*Prkdc*^*scid*^*Il2rg*^*tm1Wjl*^*/SzJ*, RRID: IMSR_JAX:005557, purchased from Charles River Laboratories) were housed at a density of 5 animals per individually ventilated cage with *ad libitum* access to food and water in a specific pathogen–free unit. Mice were administered 5 × 10^5^ cells in D-PBS via an intravenous injection to the lateral tail vein. Cells were administered in a blinded and randomized manner between cages, and after 90 minutes luciferase signal was determined to confirm successful injection with an IVIS imager (PerkinElmer). Mice were administered 120 mg/kg D-luciferin (Source BioScience, prepared in D-PBS) via an intraperitoneal injection, anesthetized with isoflurane after 5 minutes, and then placed in IVIS for image acquisition (120-second image capture). Images were collected on day 12 after intravenous dosing and at 7-day intervals thereafter up to day 75. For the treatment with PT2385, cells were prepared and dosed as described above, and IVIS was imaging performed after 90 minutes and 9 days. The images were processed, and mice were randomized to vehicle or PT2385 treatment based on total flux. PT2385 (MedChemExpress) was prepared in DMSO, and mice were administered with 10 mg/kg PT2385 (formulated as 10% DMSO and 90% of 20% sulfobutylether-β-cyclodextrin [SBE-β-CD] in saline) twice per week via an intraperitoneal injection. Control animals were treated with the vehicle (10% DMSO and 90% of 20% SBE-β-CD in saline). Images were collected twice per week as described above, and the experiment was terminated after 42 days. All experiments were performed according to protocols approved by the UK Home Office regulations and UK Animals (Scientific Procedures) Act 1986 and were approved by the University of Cambridge Animal Welfare and Ethical Review Board.

#### Statistical analyses

Quantification and data analysis of experiments are expressed as mean ± SD and *P* values calculated using analysis of variance (ANOVA) or two-tailed Student *t* test for pairwise comparisons. All statistical analyses were performed using GraphPad Prism v.8. Qualitative experiments were repeated independently to confirm accuracy.

### Statistical analysis of CAM assay

Distribution of the data was assessed using the Shapiro–Wilk test and analyzed by parametric or nonparametric tests as appropriate. *P* values less than 0.05 were considered significant.

## Results

### SET1B interacts with the RNA Pol II complex and requires multiple functional domains to regulate HIF activity

Our previous studies identified SET1B as a selective mediator of HIF target gene expression in hypoxia (Supplementary Fig. S1A; ref. 24). Although loss of SET1B reduced H3K4me3 levels at specific HIF targets, transcriptional changes were not always proportional to the reduction in H3K4me3 levels, suggesting additional roles for SET1B beyond its methyltransferase activity. We therefore set out to determine whether other SET1B functional domains contribute to HIF transcriptional regulation.

**Figure 1.**
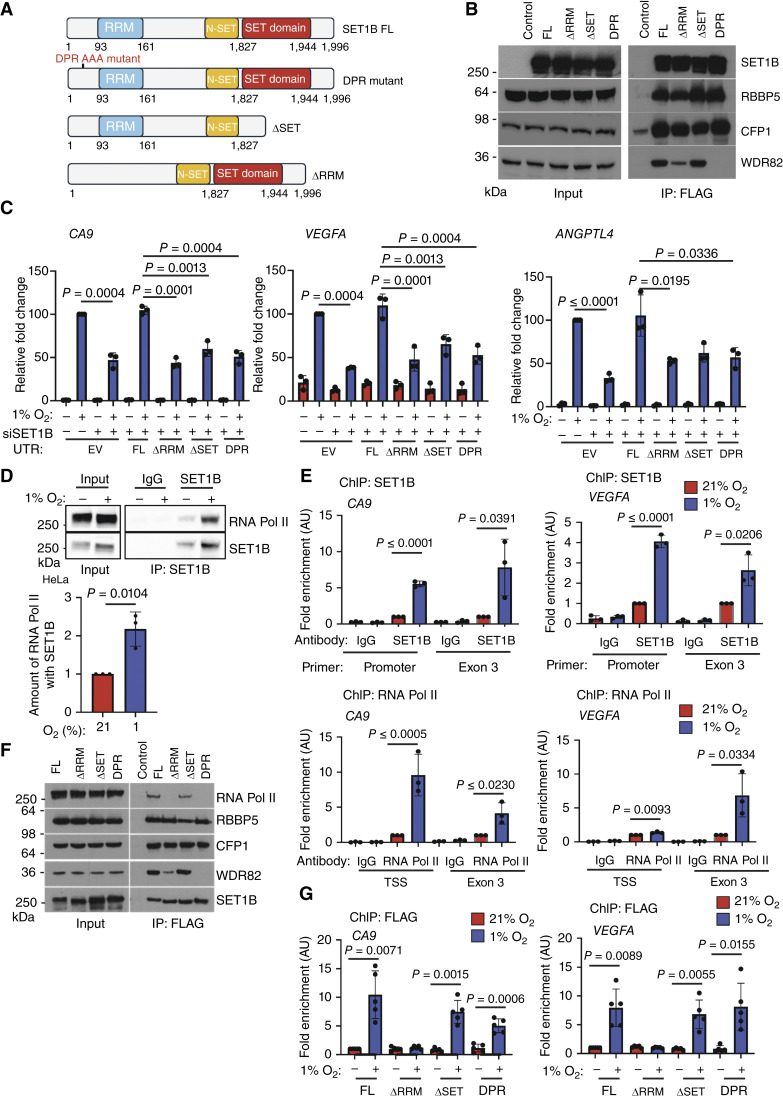
SET1B interacts with the RNA Pol II complex and requires multiple functional domains to coordinate HIF activity. **A,** Schematic representation of full-length (FL) SET1B and truncation mutants. Key functional domains are highlighted, including the SET domain (associated with methylation activity), RRM, and DPR motif (involved in RNA Pol II binding). **B,** HEK293T cells were transfected with 4 µg of the indicated expression constructs for 48 hours. Constructs were immunoprecipitated using FLAG-tagged magnetic beads, and their interaction with other members of the SET complex was evaluated by immunoblotting. **C,** A549 cells stably expressing full-length SET1B or SET1B truncation mutants were transfected with an SET1B siRNA (siSET1B) targeting the 3′-UTR and incubated under 21% or 1% O_2_ for 24 hours. mRNA expression of HIF target genes (*CA9*, *VEGFA*, and *ANGPTL4*) was assessed using qPCR. Graphs represent the mean ± SD from 3 biological replicates. Statistical analysis was performed using two-way ANOVA. EV; empty vector. **D,** Coimmunoprecipitation of SET1B and RNA Pol II. SET1B was immunoprecipitated from HeLa cells incubated under 21% or 1% O_2_ conditions for 6 hours. Samples were analyzed by immunoblotting with the indicated antibodies. Results are representative of 3 biological replicates. Graph depicts densitometric analysis of 3 independent experiments quantifying the interaction between SET1B and RNA Pol II under normoxic (21% O_2_) and hypoxic (1% O_2_) conditions. **E,** ChIP-PCR analysis of SET1B and RNA Pol II in A549 cells exposed to 21% or 1% O_2_ for 6 hours. Primers targeted the promoter and exon 3 regions of the *CA9* and *VEGFA* genes. Data represent the mean ± SD from 3 biological replicates. Statistical significance was determined using two-way ANOVA. TSS; transcriptional start site. **F,** HEK293T cells were transfected with 4 µg of the indicated expression constructs for 48 hours. Constructs were immunoprecipitated using FLAG-tagged magnetic beads, and their interaction with other members of the SET complex and RNA Pol II was assessed by immunoblotting. **G,** ChIP-PCR analysis of FLAG-tagged SET1B and SET1B mutants in A549 cells exposed to 21% or 1% O_2_ for 6 hours. Primers targeted the promoters of the *CA9* and *VEGFA* genes. Data represent the mean ± SD from 3 biological replicates. Statistical significance was determined using two-way ANOVA.

SET1B is a member of the evolutionarily conserved family of H3K4 methyltransferases that includes MLL1-4 and SET1A (also referred to as the COMPASS family; refs. 28–30). Each methyltransferase forms large complexes that share core subunits ASH2L, RBBP5, WDR5, and DPY30 to modify histone tails. Distinctively, SET1A and SET1B contain additional regulatory subunits, CFP1 (CXXC1) and WDR82, which aid in promoter recruitment and facilitate interactions with the RNA Pol II complex, respectively (31, 32). SET1B also comprises internal functional domains: the catalytic SET domain for H3K4me3 deposition, an uncharacterized RNA-recognition motif (RRM), and a trio of amino acids (aspartic acid, proline, arginine [DPR]) motif that mediates RNA Pol II interaction via WDR82 (Fig. 1A; refs. 33, 34). We therefore generated mutants deficient in these SET1B functional regions (ΔRRM, ΔSET, and a DPR to AAA mutant) and expressed these in HEK293T cells to (i) examine whether an SET1 complex can still form and (ii) determine if these SET1 mutants alter HIF transcriptional signaling.

Immunoprecipitation experiments revealed that the ΔSET mutation did not impair SET1B’s ability to form a functional complex. In contrast, the ΔRRM reduced interactions between SET1B and WDR82, whereas the DPR mutant abolished binding to WDR82 (Fig. 1B), consistent with prior findings (34). Notably, interactions with other core subunits, such as CFP1 and RBBP5, remained intact in these mutants, demonstrating that loss of these domains did not affect SET1B’s ability to form a functional complex.

To investigate the functional significance of these SET1B mutants in regulating HIF activity, we depleted endogenous SET1B (using an siRNA targeting its 3′-UTR) and stably expressing WT or SET1B mutants in A549 lung adenocarcinoma cells (Fig. 1C; Supplementary Fig. S1B–S1D). Depletion of endogenous SET1B reduced hypoxic induction of the HIF target genes (*CA9*, *VEGF*, and *ANGPTL4*), and the siRNA targeting the *SET1B* 3′-UTR reduced SET1B levels comparable with an siRNA pool targeting the SET1B coding sequence without altering the transcript level of *SET1A* (Supplementary Fig. S1C and S1D). Reconstitution with WT SET1B resulted in expression levels approximately 10-fold higher than those of endogenous SET1B (Supplementary Fig. S1D). Exogenous SET1B restored expression of the HIF target genes under hypoxia, consistent with its requirement for facilitating HIF target gene expression, whereas all the SET1B mutants failed to rescue HIF transcriptional activity (Fig. 1C).

We next explored whether the association between RNA Pol II and SET1B was altered by these different SET1B mutations, given that the DPR motif mediates SET1B’s interaction with RNA Pol II (34). Endogenous SET1B interacted with the RNA Pol II complex subunit Rbp1 under both normoxia (21% O_2_) and hypoxia (1% oxygen) in HeLa cells (Fig. 1D); however, interestingly, this interaction was enhanced under hypoxic conditions, potentially reflecting increased chromatin recruitment of SET1B from the cytoplasm in hypoxia (24, 35). ChIP assays revealed that SET1B was not only present at gene promoters and that both SET1B and RNA Pol II bound the gene bodies of HIF target genes (*CA9* and *VEGFA*) under hypoxia (Fig. 1E), consistent with SET1B associating with RNA Pol II during transcriptional elongation. The interaction between RNA Pol II and SET1B was dependent on the DPR motif, as expected (34), with the DPR mutant completely disrupting SET1B’s association with both RNA Pol II and WDR82 (Fig. 1F). Loss of the RRM also reduced interaction with RNA Pol II (Fig. 1F), suggesting that the RRM domain plays a critical role in stabilizing the interaction between RNA Pol II and SET1B through WDR82. However, the ΔSET mutation did not disrupt RNA Pol II binding (Fig. 1F) but was still required to restore HIF-dependent transcription (Fig. 1C). Therefore, these findings indicate that both the SET domain and the ability of SET1B to bind to RNA Pol II are important for the regulation of HIF-mediated transcription.

We considered whether defective HIF activity in the SET1B mutants reflects impaired chromatin engagement. We therefore performed FLAG-ChIP for WT SET1B and mutants less than 21% and 1% O_2_ at the *CA9* and *VEGFA* promoters (Fig. 1G). As expected, WT SET1B was robustly recruited in hypoxia (1% O_2_). Notably, loss of the SET domain or mutation of the DPR motif did not reduce chromatin binding, indicating that catalytic activity is not required for recruitment; rather, the associated methyltransferase function and coupling to Pol II likely govern transcriptional output downstream of occupancy. By contrast, deletion of the RRM motif abolished hypoxia-induced recruitment, suggesting that RNA binding may be critical for stabilizing SET1B–chromatin interactions (Fig. 1G). This requirement for RNA engagement is consistent with observations from the yeast SET complex (36).

### SET1B is required for sustained HIF2-dependent activity in ccRCC

As SET1B binding was detected within the gene body of HIF target genes, we hypothesized that SET1B may be equally important for sustaining HIF activity after HIF binding and initiation of the hypoxic response. To explore this, we utilized ccRCC as a model of sustained HIF activation caused by the loss of VHL function (37, 38). Of note, ccRCC typically demonstrates sustained HIF2 activation, as HIF1 is frequently deleted as an early tumor-initiating event (39).

**Figure 2.**
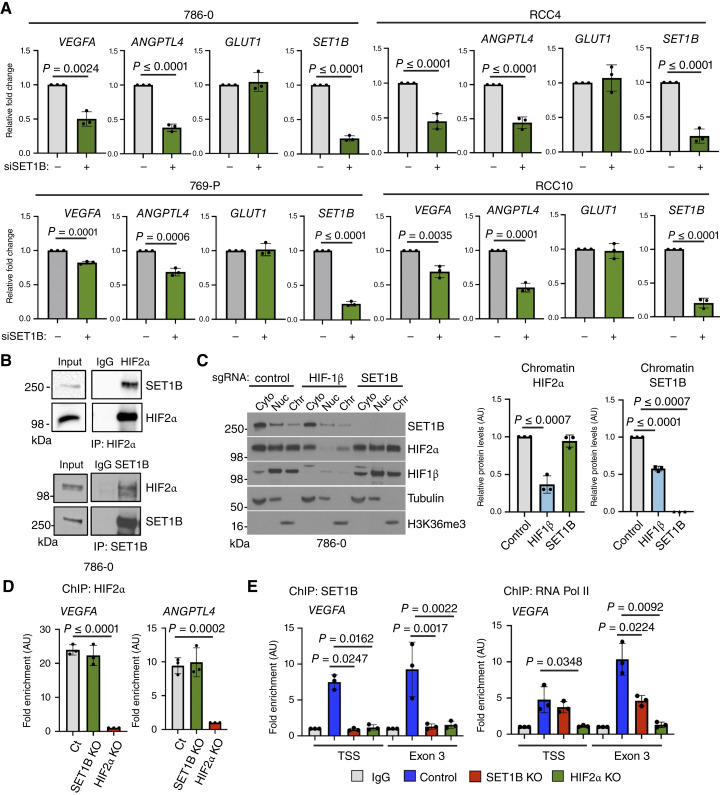
SET1B is required for sustained HIF activity. **A,** 786-0, RCC4, 769-P, and RCC10 ccRCC cell lines were transfected with control or SET1B siRNA (siSET1B) for 48 hours. The expression of HIF target genes and *SET1B* was assessed using qPCR. Graphs are representative for 3 biological replicates and depict the mean ± SD. Two-way ANOVA. **B,** Endogenous HIF2α or SET1B was immunoprecipitated from 786-0 cells and immunoblotted. Immunoblot is representative of 3 biological replicates. **C,** 786-0 cells were fractionated into cytoplasmic (Cyto), nucleoplasm (Nuc), and chromatin (Chr) fractions and immunoblotted for SET1B and HIF2α. The ratio of SET1B and HIF2α in the chromatin fraction was quantified using ImageJ (*n* = 3 biologically independent experiments; mean ± SD). **D,** ChIP-PCR of HIF2α in WT, SET1B KO, and HIF2α KO 786-0 cells. ChIP-PCR was performed using primers targeting the promoters of *VEGFA* and *ANGPTL4.* Graphs are representative for 3 biological replicates and depict the mean ± SD. Two-way ANOVA. **E,** ChIP-PCR of SET1B and RNA Pol II in WT, SET1B, and HIF2α KO 786-0 cells. ChIP-PCR was performed using primers targeting the promoter and exon 2 of *VEGFA* (*n* = 3 biological replicates). Graphs show mean ± SD. Two-way ANOVA. IP, immunoprecipitation.

We depleted SET1B in a panel of ccRCC cell lines (786-0, 769-P, RCC4, and RCC10) using siRNA and measured the expression of HIF target genes (Fig. 2A). SET1B depletion in all cell lines led to a selective reduction in HIF target gene expression, with effects on *VEGFA* and *ANGPTL4* but not *GLUT1*, as expected (24). Similar results were obtained using shRNA- and CRISPR-mediated depletion of SET1B in 786-0 cells (Supplementary Fig. S2A and S2B). Importantly, loss of SET1B did not affect the mRNA or protein levels of HIF2α across a panel of ccRCC cell lines, highlighting that SET1B regulates HIF2α-dependent transcriptional activity at the chromatin level (Supplementary Fig. S2A–S2E). Although SET1B depletion reduced HIF-dependent transcription across all cell lines, the effect was less pronounced in 769-P cells (Fig. 2A). Such differences in sensitivity are to be expected and are consistent with differential responses of ccRCC cells to HIF2α inhibition (40). We also confirmed that the effect of SET1B depletion was HIF dependent as HIF2α KO cells showed no further reduction in HIF target gene transcription in the absence of SET1B (Supplementary Fig. S2F). Moreover, SET1B overexpression in HIF2α KO cells could not rescue HIF2 target expression, further substantiating that the effect of SET1B loss is dependent on HIF2 (Supplementary Fig. S2G). Immunoprecipitation experiments confirmed that endogenous SET1B interacts with HIF2α in 786-0 cells (Fig. 2B). Furthermore, we mapped the interaction between HIF2α and SET1B, revealing that SET1B preferentially binds to the C-terminus of HIF2α, specifically interacting with the PAS-B domain and the C-terminus, with no detectable interaction with the N-terminus, which includes the basic helix–loop–helix and PAS-A domains (Supplementary Fig. S2H).

Our previous work demonstrated that under hypoxic conditions, SET1B relocalizes from the cytosol to chromatin in a HIF-dependent manner (24). However, whether sustained HIF activation alters the subcellular localization of HIF2α and SET1B had not been determined. To address this, we performed subcellular fractionation in WT, HIF1β, or SET1B KO 786-0 cells. We found that SET1B was distributed between the cytoplasm, nucleus, and chromatin, similarly to the distribution of HIF2α (Fig. 2C). Loss of HIF1β, which impedes HIF2α binding to chromatin, resulted in a reduction of both HIF2α and SET1B in the chromatin fraction, suggesting that SET1B binding to chromatin in ccRCC is partially dependent on HIF2α (Fig. 2C). However, SET1B deficiency did not prevent the chromatin localization of HIF2α, consistent with our prior observations with HIF1α (24). HIF2α KO cells also had less chromatin-bound SET1B (Supplementary Fig. S2I).

To further determine if SET1B loss altered HIF2α binding to HIF target genes, we performed ChIP-PCR for HIF2α in WT, SET1B KO, or HIF2α KO 786-0 cells, using primers targeting the promoters of *VEGFA* and *ANGPTL4*. Loss of SET1B did not affect HIF2α binding to target gene loci, confirming that SET1B-dependent regulation of HIF2α activity occurs at the chromatin level (Fig. 2D). Both SET1B’s methyltransferase activity and binding to RNA Pol II seemed to be important for the regulation of HIF target genes, as loss of either SET1B or HIF2α reduced H3K4me3 levels at the *VEGFA* promoter (Supplementary Fig. S2J) and resulted in reduced RNA Pol II abundance at exon 3 of *VEGFA* (Fig. 2E). These results highlight the requirement for SET1B in driving transcription at HIF2α target genes, likely dependent on both methyltransferase activity and RNA Pol II recruitment.

### SET1B facilitates HIF2-driven ccRCC progression

**Figure 3.**
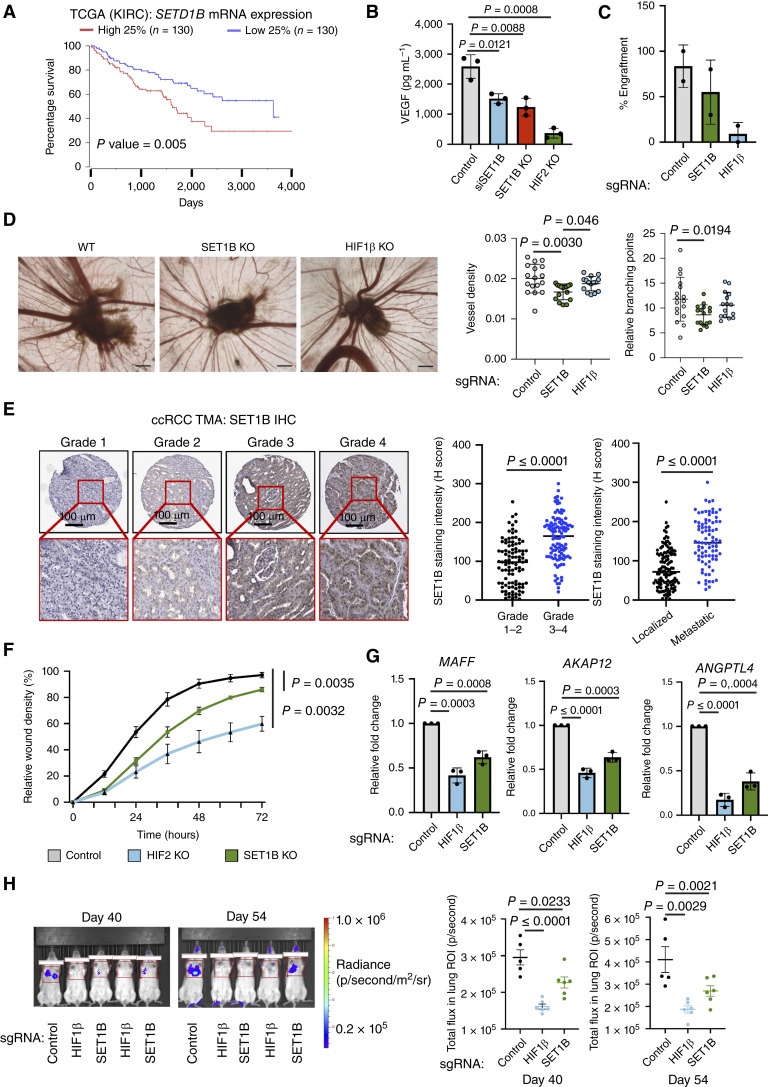
SET1B is required for sustained HIF2-dependent activity in ccRCC. **A,** High expression of SETD1B (*SET1B* gene name) in kidney cancer is associated with poor outcomes. Kaplan–Meier survival analysis of TCGA data for ccRCC, comparing tumors in the highest and lowest quartiles of SETD1B mRNA expression. *n* = 130 patients for each group. Log-rank test. **B,** VEGFA ELISA in control, SET1B depletion, SET1B KO, and HIF2α KO 786-0 cells. Cells were incubated for 48 hours and before supernatants were collected. The graph is representative of 3 biological replicates. Two-way ANOVA. **C,** Graphs showing the percentage engraftment of control, HIF1β, and SET1B KO cells in the CAM assay. **D,** Representative images of 786-O parental (*n* = 17), SET1B KO (*n* = 16), and HIF1β KO (*n* = 15) CAM assay. The xenografts and surrounding CAM was fixed *in ovo* to preserve blood in the vessels and then imaged from the underneath after dissection. Scale bars, 1 mm. Vessel density was calculated as total vessel length (pixels)/total area analyzed (pixels^2^) per ROI. Brown–Forsythe ANOVA test, F(2,33.38) = 7.156, *P* = 0.0026. Dunnett T3 multiple comparison test; parental (P) vs. SET1B KO (S), *P* = 0.0076; parental vs. HIF1β KO (H), *P* = 0.4873; SET1B KO vs. HIF1β KO, *P* = 0.0136. **D,** Branching points relative to area analyzed (mm^2^). Ordinary one-way ANOVA, F(2, 45) 4.019, *P* = 0.0248. Tukey multiple comparison test; parental vs. SET1B *P* = 0.0076; parental vs. HIF1β *P* = 0.4873; SET1B vs. HIF1β *P* = 0.0136. **E,** IHC of SET1B was performed on a ccRCC tissue microarray (TMA). Samples were assessed and graded 1–4 with 1 being the least aggressive and grade 4 being the most aggressive. SET1B staining intensity across the tumor was calculated as an H score which accounts for the staining intensity and the % of positive cells detected. Samples were subdivided based on grade, and SET1B intensity was plotted. Significance was assessed using a two-way ANOVA. **F,** Control, HIF2α KO, and SET1B KO 786-0 cells were embedded in Matrigel, and cellular invasion was measured over indicated time using Incucyte. Graphs are representative for 3 biological replicates and depict the mean ± SD. Two-way ANOVA. **G,** qPCR of HIF targets associated with metastasis (*MAFF*, *AKAP12*, and *ANGPTL4*) in 786-0 cells depleted of HIF1β and SET1B using CRISPR (*n* = 3 biologically independent samples, mean ± SD). **H,** ccRCC mouse xenograft model. WT, HIF1β, and SET1B-depleted 786-0 cells expressing luciferase were injected into the tail vein of nude mice. Bioluminescence was measured and quantified from the lungs on day 40 and day 54 (control = 5; HIF1β KO = 6; and SET1B KO = 6). Mean ± SD. Two-way ANOVA. ROI, region of interest; siSET1B, SET1B siRNA.

To investigate the functional role of SET1B in ccRCC progression, we began by analyzing TCGA database (https://www.cancer.gov/tcga). Specifically, we examined the mRNA expression levels of SET1B in the Kidney Renal Clear Cell Carcinoma (KIRC) ccRCC dataset and assessed its correlation with patient survival (Fig. 3A). Patients with the highest *SET1B* mRNA expression (top 25%) had a significantly lower survival rate than those with the lowest *SET1B* expression (Fig. 3A). A positive correlation between *VEGFA* and *SET1B* mRNA expression was identified using cBioPortal (https://www.cbioportal.org/; Supplementary Fig. S3A). Consistently, SET1B loss reduced VEGF secretion in 786-O cells, indicating that SET1B is a key modulator of HIF-driven tumor angiogenesis in ccRCC, consistent with our previous observations (Fig. 3B; ref. 24).

To further validate the role of SET1B in HIF-mediated angiogenesis, we employed a CAM assay, which utilizes the highly vascularized membrane of a developing chick embryo as a physiologic model of angiogenesis. The CAM provides a rich blood supply and an immunodeficient environment, allowing quantification of vascularization following cell engraftment (41–44). Vascularization was calculated by analyzing regions of microvessels in between larger vessels using IKOSA CAM Assay software (Supplementary Fig. S3B).

WT, HIF1β KO, and SET1B KO 786-O mixed-population cells were engrafted onto the CAM, and angiogenesis was quantified. A substantial proportion of HIF1β KO cells failed to engraft, underscoring the requirement for HIF in tumor establishment and growth in ccRCC (Fig. 3C), consistent with previous murine xenograft data showing that HIF1β-deficient 786-0 cells failed to form tumors (24). SET1B KO cells engrafted to a similar amount to the WT, but SET1B depletion resulted in significantly reduced vessel density and branching complexity, processes which are known to be highly dependent on *VEGFA* (44). The CAM assay therefore corroborates SET1B as a critical regulator of HIF-dependent angiogenesis *in vivo* (Fig. 3D)**.**

To further investigate if SET1B is correlated with ccRCC progression, we examined SET1B levels using IHC in a clinical cohort of ccRCC samples (*n* = 317; Fig. 3E; ref. 27). SET1B levels were calculated using an H score (0–300), which encompasses the intensity of staining and the number of positive cells. Increased SET1B levels within the tumor correlated with high tumor grade and in patients that had metastatic disease (Fig. 3E).

To understand the functional requirement for SET1B in controlling HIF2 activity in ccRCC, we focused on *in vitro* assays of metastatic potential, as we had previously demonstrated that SET1B loss reduced tumor establishment and growth in a 786-0 xenograft model, and assessed the impact of SET1B loss on cell migration and invasion using a scratch and invasion assay (24). Depletion of SET1B or treatment with the HIF2 inhibitor (PT2385) in 786-0 cells had no effect on cell migration *in vitro* (Supplementary Fig. S3C). However, loss of HIF2α reduced cellular invasion, confirming the role of HIF2 in cellular invasion. Similar results were also observed for SET1B depletion, although not to the same extent as HIF2α KO (Fig. 3F). SET1B depletion also reduced the HIF2-mediated transcription of several genes known to be determinants of metastases (*MAFF*, *AKAP12*, and *ANGPTL4*; Fig. 3G; ref. 45), suggesting that SET1B coordinates cellular metastasis as well as angiogenesis in ccRCC.

Lastly, we explored the biological role of SET1B in ccRCC using an experimental *in vivo* metastasis model which measures the cell’s ability to extravasate and colonize the lung. SET1B and HIF1β were depleted in 786-0 luciferase-expressing cells using CRISPR before the cells were injected into the tail vein of nude mice (Supplementary Fig. S3D and S3E). Cellular metastasis was determined by quantifying luciferase levels within the lung on days 40 and 54 (Fig. 3H). Loss of HIF1β or SET1B resulted in very low detection of lung luciferase signal (Fig. 3H). Collectively, these findings indicate that SET1B helps drive angiogenesis and metastatic disease in an HIF2-dependent manner.

### Depletion of SET1B potentiates HIF2 inhibition in ccRCC

**Figure 4.**
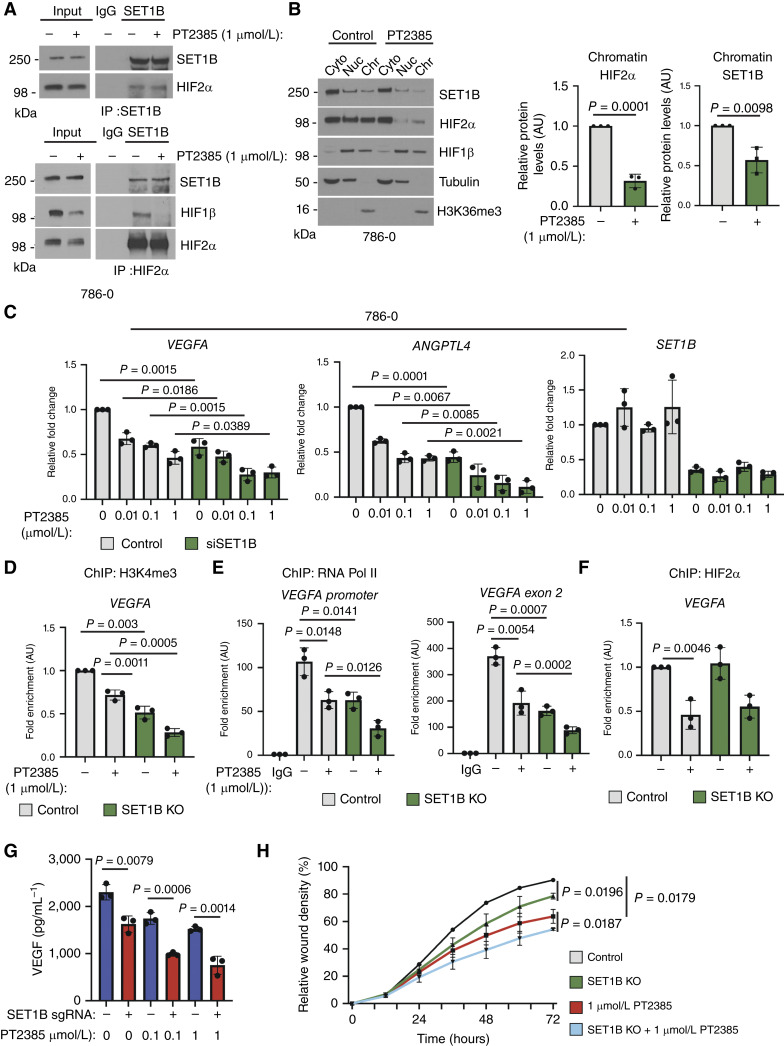
Depletion of SET1B potentiates HIF2 inhibition in ccRCC. **A,** WT 786-0 cells were treated with and without 1 µmol/L PT2385 for 24 hours. Endogenous SET1B and HIF2α were immunoprecipitated, and their interaction was evaluated by immunoblotting using the indicated antibodies. Immunoblots are representative of 3 biological replicates. **B,** 786-0 cells were treated with and without 1 µmol/L PT2385, fractionated into cytoplasmic (Cyto), nucleoplasm (Nuc), and chromatin (Chr) fractions, and SET1B and HIF2α were monitored using immunoblotting. The ratio of SET1B and HIF2α in the chromatin fraction was quantified using ImageJ (*n* = 3 biologically independent experiments, mean ± SD). **C,** WT 786-0 cells were transfected with a control and an SET1B siRNA (siSET1B) for 48 hours. Twenty-four hours prior to harvesting, cells were treated with indicated concentrations of PT2385. HIF2α activity was determined by performing qPCR for *VEGFA* and *ANGPTL4* (*n* = 3 biologically independent experiments, mean ± SD). SET1B mRNA levels was analyzed to validate successful depletion. ChIP-PCR of H3K4me3 (**D**), RNA Pol II (**E**), and HIF2α (**F**) in WT and SET1B KO 786-0 cells treated in the presence and absence of 1 µmol/L PT2385. ChIP-PCR was performed using primers surrounding the *VEGFA* promoter (*n* = 3 biological replicates). Graphs show mean ± SD. Two-way ANOVA. **G,** VEGFA ELISA in control and SET1B-depleted 786-0 cells. Twenty-four hours before collecting supernatants, cells were treated with 1 µmol/L PT2385. Graph is representative of 3 biological replicates. Two-way ANOVA. **H,** Control and SET1B KO 786-0 cells treated with and without 1 μmol/L PT2385 were embedded in Matrigel, and cellular invasion was measured over indicated time using Incucyte. Graphs are representative for 3 biological replicates and depict the mean ± SD. Two-way ANOVA.

PT2385 (belzutifan) is the first selective small-molecule inhibitor of HIF2α activity in clinical use and inhibits the interaction between HIF2α and HIF1β (Supplementary Fig. S4A; ref. 46). Given that PT2385 does not fully inhibit HIF2 activity, we hypothesized that depleting SET1B could enhance PT2385-mediated suppression of HIF2 signaling. Consistent with this notion, immunoprecipitation studies in 786-0 cells, treated and untreated with PT2385, revealed that although PT2385 disrupts the interaction between HIF1β and HIF2α, it does not affect the interaction between HIF2α and SET1B (Fig. 4A), nor does it alter the protein levels of SET1B (Supplementary Fig. S4B). Moreover, cellular fractionation analyses revealed that PT2385 did not fully prevent HIF2 or SET1B from binding chromatin (Fig. 4B), consistent with some residual HIF2 transcriptional activity remaining following PT2385 treatment. Therefore, to test if SET1B loss could enhance the activity of HIF2 inhibition, we used siRNA-mediated SET1B depletion followed by treatment with different concentrations of PT2385. SET1B deficiency enhanced suppression of HIF2-mediated transcription (Fig. 4C). This combinatorial effect was also observed using CRISPR-mediated depletion of SET1B and with two different SET1B shRNAs (Supplementary Fig. S4C–S4E). Importantly, the combination of SET1B depletion with PT2385 enabled the use of lower PT2385 doses, leading to a more pronounced downregulation of HIF2 activity (Fig. 4C). A similar additive effect was observed in 769-P cells (Supplementary Fig. S4F). In contrast, RCC4 and RCC10 cells, which express both HIF1α and HIF2α, exhibited reduced HIF activity with SET1B depletion alone, without additional benefit from PT2385, suggesting potential compensatory mechanisms played by HIF1α in these cells (Supplementary Fig. S4G and S4H).

Mechanistically, concurrent SET1B loss and HIF2α inhibition markedly reduced H3K4me3 levels at the promoter coupled with reduced binding of RNA Pol II levels at both the promoter and exon 2 of *VEGFA*, surpassing the effects observed with SET1B KO or PT2385 treatment alone (Fig. 4D and E). Notably, SET1B depletion did not alter HIF2α binding in isolation or in combination with HIF2 inhibition, consistent with SET1B modulating residual HIF2α transcriptional activity on the chromatin (Fig. 4F).

To further establish the functional significance of the synergy between SET1B depletion and HIF2α inhibition, we evaluated VEGF secretion and cellular invasion in SET1B-depleted 786-0 cells following HIF2 inhibition. PT2385 treatment significantly reduced VEGF secretion, with the combination of SET1B depletion and PT2385 resulting in a more substantial reduction compared with control cells (Fig. 4G). This additive effect was similarly observed in cellular invasion assays, in which SET1B loss alone decreased cellular invasion, but the combination with PT2385 had an enhanced inhibitory effect (Fig. 4H).

### Targeting SET1B to circumvent HIF2 inhibitor resistance

**Figure 5.**
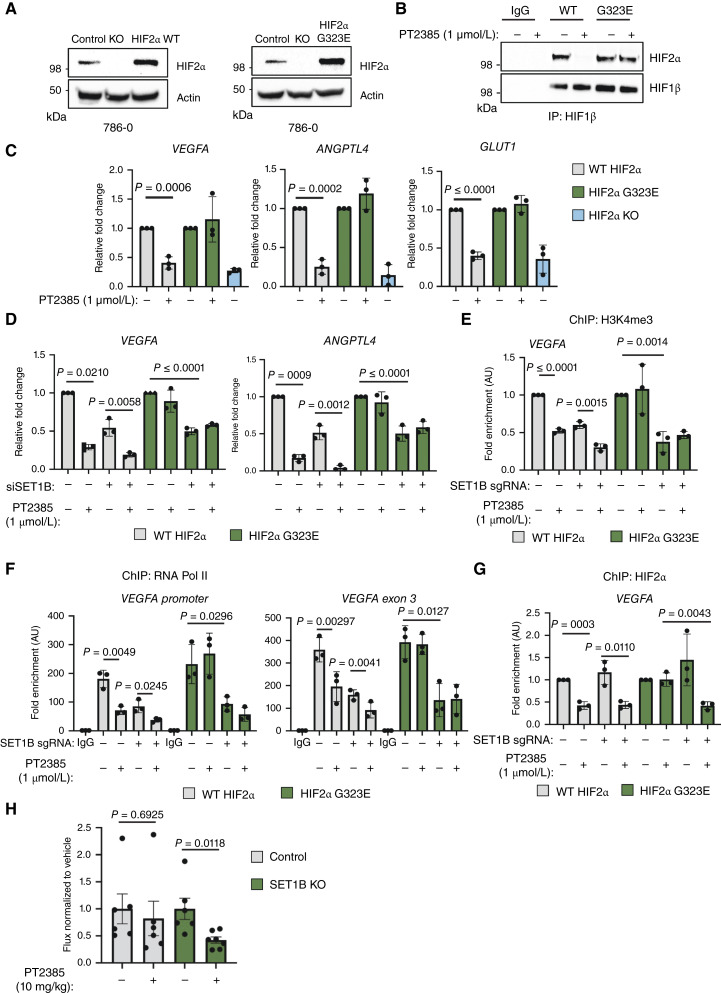
SET1B depletion overcomes resistance to HIF2 inhibition in ccRCC. **A,** Immunoblots of WT, HIF2α KO, and HIF2α KO 786-0 cells reconstituted with WT HIF2α or the HIF2α G323E mutation. **B,** HIF2α KO cells reconstituted with WT and HIF2 G323E mutation were treated with 1 µmol/L  PT2385 for 24 hours. Endogenous HIF1β was immunoprecipitated, and the interaction with HIF2α was assessed by immunoblotting. Immunoblot is representative of 3 independent experiments. **C,** HIF2α KO cells reconstituted with WT and HIF2 G323E mutation were treated with 1 μmol/L PT2385 for 24 hours. HIF2α target gene and *SET1B* mRNA expression was assessed by qPCR (*n* = 3 biological replicates). Graphs show mean ± SD. Two-way ANOVA. **D,** HIF2α KO cells reconstituted with WT and HIF2α G323E mutation were transfected with an SET1B siRNA for 48 hours. Twenty-four hours before harvesting, cells were treated with and without 1 μmol/L PT2385. qPCR analysis was performed using primers targeting the *VEGFA* and *ANGPTL4* mRNAs (*n* = 3 biological replicates). Graphs show mean ± SD. Two-way ANOVA. **E,** ChIP of H3K4me3 and RNA Pol II (**F**) in HIF2α KO cells reconstituted with WT and HIF2α G323E mutation following CRISPR-mediated SET1B depletion 24 hours before harvesting cells were treated with 1 μmol/L PT2385. ChIP-PCR was performed using primers targeting the *VEGFA* promoter (*n* = 3 biological replicates). Graphs show mean ± SD. Two-way ANOVA. **G,** ChIP of HIF2α in HIF2α KO cells reconstituted with WT and HIF2α G323E mutation following CRISPR-mediated SET1B depletion 24 hours before harvesting cells were treated with 1 μmol/L PT2385. ChIP-PCR was performed using primers targeting the *VEGFA* promoter and exon 3 (*n* = 3 biological replicates). Two-way ANOVA. **H,** ccRCC mouse xenograft model. WT and SET1B-depleted 786-0 cells expressing luciferase were injected into the tail vein of nude mice. Bioluminescence was measured and quantified from the lungs on day 18, and luminescence was normalized to the vehicle from the specific genotype (control = 6; control + PT2385 = 6; SET1B KO = 6; and SET1B KO + PT2385 = 7). Mean ± SEM. Kruskal–Wallis test). siSET1B, SET1B siRNA.

Recent clinical studies show that patients can acquire resistance to prolonged HIF2α inhibition, a phenomenon previously linked to increased p53 activity (47). However, work from the Kaelin laboratory indicates that p53 status alone does not predict sensitivity to HIF2 blockade (48). With extended therapy, a recurrent HIF2α G323E substitution emerges within the inhibitor-binding pocket (47). The G323E mutation enables HIF2α to reassociate with HIF1β, thereby promoting ccRCC progression. Targeting SET1B may therefore provide a strategy for combating HIF2α inhibitor resistance. To explore this, we reconstituted HIF2α KO 786-0 cells with either WT HIF2α or the HIF2α G323E mutant (Fig. 5A) and examined the effects of SET1B depletion.

Reconstitution with the HIF2α G323E mutant enabled HIF2α to reassociate with HIF1β, even in the presence of PT2385 (Fig. 5B), preventing the reduction in HIF2 target gene expression (Fig. 5C). Although 786-0 cells expressing the G323E mutant were resistant to PT2385, HIF target gene transcription was still reduced following SET1B depletion (Fig. 5D). SET1B depletion led to a decrease in H3K4me3 levels and RNA Pol II levels across the *VEGFA* gene in both WT and G323E mutant cells (Fig. 5E and F), without affecting HIF2α binding (Fig. 5G), indicating that SET1B loss reduced the transcriptional activity of the gene.

**Figure 6.**
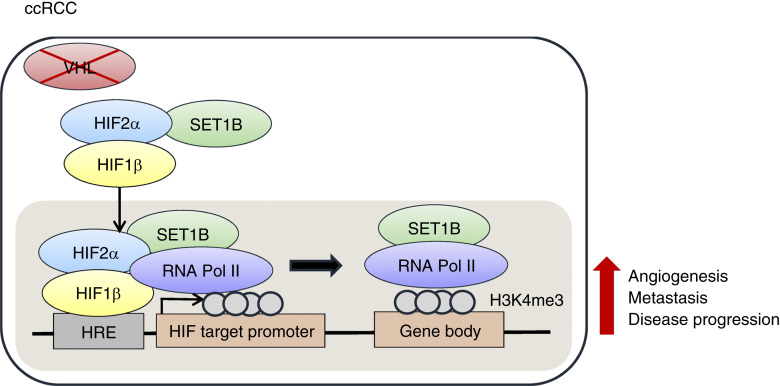
Model for the role of SET1B in ccRCC. In ccRCC, HIF2 is constitutively active because of the loss of VHL protein. SET1B is recruited to HIF target genes by the HIF complex, in which it plays a critical role in initiating and sustaining HIF transcription. This is achieved through its H3K4me3 activity and its interaction with the RNA Pol II complex. The enhanced HIF2 transcription leads to increased angiogenesis, metastasis, and disease progression.

To functionally evaluate the potential of SET1B depletion for circumventing HIF2 inhibitor resistance *in vivo*, we assessed the combination effect of SET1B depletion and submaximal HIF2 inhibition in the lung metastasis model. We first established a dose of PT2385 that would exert a modest effect on lung colonization and observed that 10 mg/kg PT2385 produced a mild reduction in the number of lung metastases. We then compared the effect of low-dose PT2385 alone or in combination with SET1B depletion. Fewer lung colonies were observed in the SET1B-depleted and low-dose PT2385–treated mice (Fig. 5H). These results suggest that SET1B could serve as a potential therapeutic target to overcome HIF2 inhibitor resistance and suppress ccRCC progression, by blocking selective HIF2-dependent transcription at the chromatin level (Fig. 6).

## Discussion

Although we initially hypothesized that SET1B primarily modulates H3K4me3 levels at HIF target loci, our findings reveal a more intricate mechanism of SET1B-dependent transcriptional regulation. Using a model of sustained HIF activation, we show that SET1B is not only essential for initiating HIF activity but also crucial for maintaining it over time. Notably, we present evidence that SET1B, in addition to its recruitment to gene promoters, binds across the gene bodies of HIF targets, with this binding closely associated with RNA Pol II occupancy.

Several studies have emphasized that H3K4me3 regulates RNA Pol II activity by facilitating the release of RNA Pol II from proximal-pause and promoting RNA Pol II elongation activity (25, 34). However, as SET1B seems to travel with the RNA Pol II complex along the DNA, it is possible that SET1B may play additional roles in transcriptional regulation. This notion would be consistent with other studies focusing on the regulation of HIF target genes, in which transcriptional complexes such as DNA-PK, FACT, TIP60, and ZMYND8 enhance HIF-dependent transcription by modulating RNA Pol II activity (15, 17, 18, 49). It will be helpful in future work to determine whether the spreading of H3K4me3 into gene bodies contributes to the regulation of RNA Pol II activity at HIF target genes, as observed for the establishment of cellular identity in embryonic stem cells (50).

Our studies on SET1B functional domains show that the DPR motif, RRM and SET domains are required for modulating HIF transcriptional activity. The involvement of the DPR motif of SET1B is consistent with prior studies, and the DPR motif has been shown to modulate transcription through binding to WDR82 in mouse embryonic stem cells (34). Although RRMs are known to bind RNA, both SET1A and SET1B each contain a single RRM, in contrast to other RNA-interacting proteins that generally require two RRMs for RNA binding (51). As loss of the RRM reduces the ability of SET1B to interact with RNA Pol II, this raises the possibility that SET1B may recognize specific RNA species or alternatively RNA binding may modulate the methylase activity of SET1B which has been observed for other enzymes (52). It will be of interest to address RNA binding by SET1B in future studies, but it is noteworthy that the yeast SET1 complex binds nascent RNA and this binding is required for the deposition of H3K4me3 (36).

The requirement of the SET domain for SET1B-mediated regulation of HIF target gene was evident in our studies, but the role of H3K4me3 in transcription remains debated. Recent studies have highlighted the importance of H3K4me3 in RNA Pol II pause release at the promoter (25), and our data suggest that SET1B methylase activity may contribute to transcriptional changes even with modest changes in H3K4me3 levels. However, it is also possible that SET1B may methylate other proteins aside from histones, just as SET1A has been proposed to monomethylate YAP, promoting YAP activity and preventing its nuclear export (53). Similarly, studies have emphasized the importance of RNA Pol II lysine methylation in transcription regulation (54, 55).

In ccRCC, loss of VHL leads to constitutive HIF activation, with several studies highlighting the importance of HIF2α in promoting angiogenesis and metastasis (56, 57). Using a panel of ccRCC-derived cell lines, we found that SET1B helps sustain HIF-dependent activity at the chromatin level. Additionally, SET1B expression correlates with disease grade and metastasis in patient samples, with particularly high levels observed in metastatic ccRCC. Furthermore, depletion of SET1B impaired the ability of ccRCC cells to survive *in vivo* in an experimental metastasis model. These findings underscore the role of SET1B in sustaining HIF2α activity and driving the expression of genes involved in angiogenesis and metastatic spread.

The development of HIF2α-specific inhibitors are a promising therapeutic approach for ccRCC, now in routine clinical practice in some nations, but HIF2α inhibition can have off-target effects due to its roles in other physiologic processes, including immune cell function and carotid body activity (58–60). Pursuing selective modulation of SET1B activity may be a potential strategy for targeting ccRCC while reducing the impact on other essential HIF2-dependent functions. Finally, resistance to HIF2α inhibitors has been observed in some patients after prolonged treatment with belzutifan. Several mechanisms have been proposed, including the upregulation of p53 activity or the G323E mutation within the inhibitor-binding pocket (47). However, these findings are debated, as a recent study by the Kaelin Jr group demonstrated that activation of the p53 pathway did not dictate the sensitivity of ccRCC to HIF2 inhibition (48). Our findings now indicate that targeting SET1B in patients with the G323E mutation could be a viable strategy to reduce HIF2-driven pathways involved in angiogenesis and metastasis. Our initial data using low-dose PT2385 show an additive effect with SET1B depletion in the lung colony formation assay, but the ultimate test will require modeling the HIF2α mutations alongside PT2385 or SET1B inhibition *in vivo*. Whether therapeutic targeting of SET1B is possible remains to be seen, but inhibitors for the SET family of methyltransferases are being actively pursued (61–63). Our studies also indicate that inhibition of SET activity alone may not be sufficient and that other domains, including those involved in the recruitment of Pol II and RNA binding, need to be considered.

## Supplementary Material

Table S1 Primer sequences for HIF-2 cloning.

Table S2 sgRNA and shRNA sequences.

Table S3 Lists of reagents and antibodies.

Table S4 qpCR and ChIP primers.

Figure S1 SET1B interacts with the RNA Pol II complex and requires multiple functional domains to coordinate HIF activity.

Figure S2 SET1B is required for sustained HIF-2 activity.

Figure S3 The effect of SET1B depletion on VEGF expression and on cell migration in ccRCC.

Figure S4 Loss of SET1B enhances the efficacy of HIF-2 Inhibition.

## Data Availability

The mRNA expression data for SET1B in KIRC were obtained from TCGA database, more specifically OncoLnc (http://www.oncolnc.org/), and analyzed by comparing the top 25% and bottom 25% of SET1B mRNA expression levels within the patient cohort. All raw data generated during this study are available from the corresponding author upon reasonable request.
