# Supplementary material for: SET1B Drives Sustained HIF Activity and Disease Progression in Clear-Cell Renal Cell Carcinoma
Source: Cancer Res. 2026 Apr 6;86(15):3704–19. doi: 10.1158/0008-5472.CAN-25-1076 (PMC13434302; doi:10.1158/0008-5472.CAN-25-1076)
Supplement: Supplementary file 1 [file can-25-1076_figure_s1_suppsf1.pdf]

Figure S1: SET1B interacts with the RNA Pol II complex and relies on multiple functional domains to regulate HIF activity.

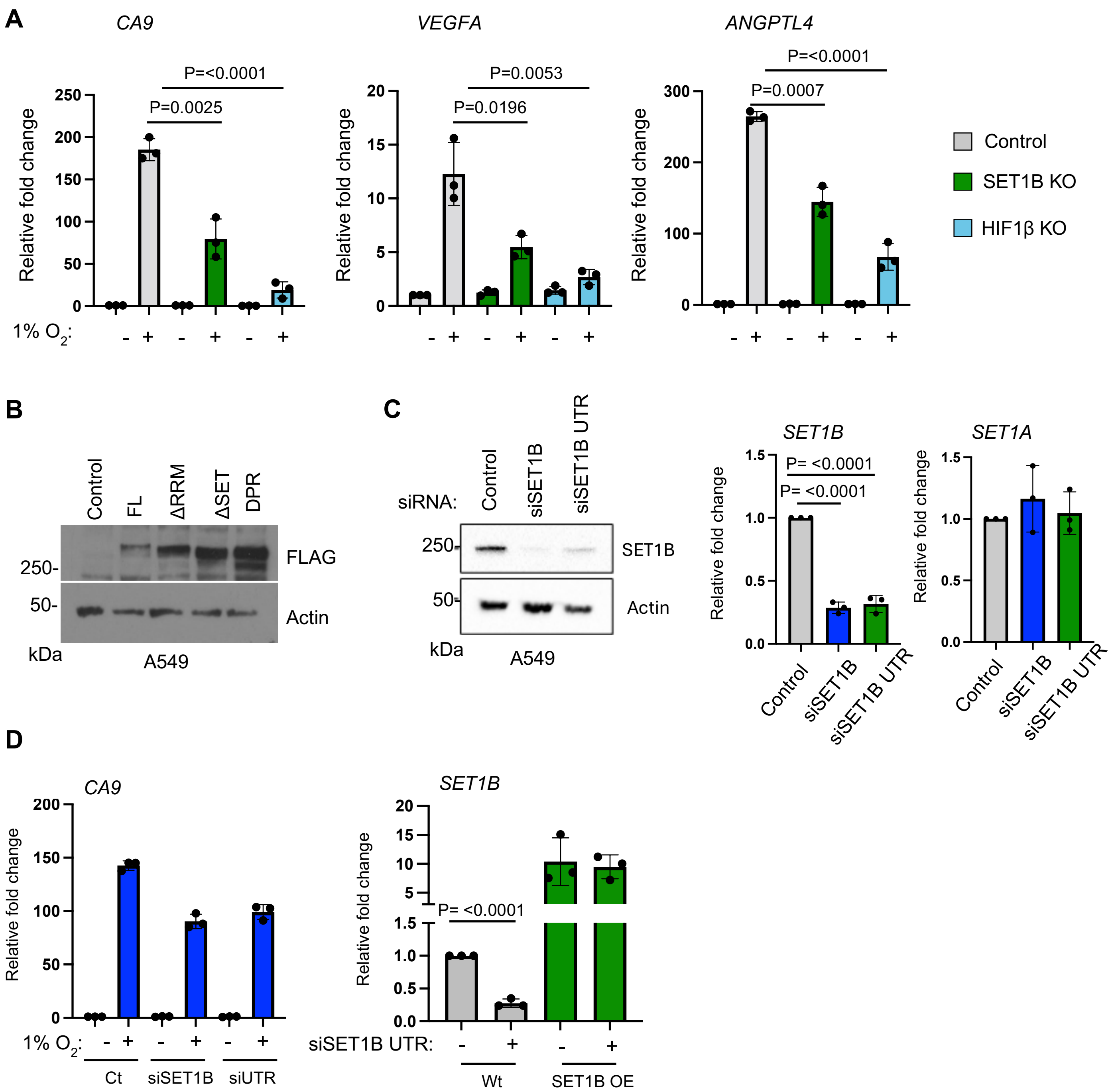

Figure S1. SET1B interacts with the RNA Pol II complex and requires multiple functional domains to coordinate HIF activity.

(A) qPCR analysis of HIF target genes. A549 cells were transduced with sgRNAs targeting HIF1β and SET1B. Cells were incubated at 21% or 1% oxygen for 24 hours before qPCR was performed to analyse the mRNA expression levels of HIF target genes CA9, VEGFA and ANGPTL4 (n= 3 biological replicates). Graphs show mean ± SD. Two-way ANOVA. **(B)** Immunoblot analysis of A549 cells stably expressing full-length SET1B and various SET1B truncation mutants**. (C)** A549 cells were transfected with a pool of SET1B siRNAs and an siRNA targeting the 3'UTR of SET1B for 48 hrs. Samples were assessed using immunoblotting and qPCR using primers targeting *SET1B* and *SET1A*. Graphs are representative for 3 biological replicates and depict the mean ± SD. Two-way ANOVA. **(D)** A549 cells were transfected with a pool of SET1B siRNAs and an siRNA targeting the 3'UTR of SET1B for 48 hrs. 24 hrs before sample collection cells were incubated at 21% and 1% oxygen for 24 hrs. qPCR was performed using primers targeting the CA9 and SET1B. Graphs are representative for 3 biological replicates and depict the mean ± SD. Two-way ANOVA.
