# Supplementary material for: SET1B Drives Sustained HIF Activity and Disease Progression in Clear-Cell Renal Cell Carcinoma
Source: Cancer Res. 2026 Apr 6;86(15):3704–19. doi: 10.1158/0008-5472.CAN-25-1076 (PMC13434302; doi:10.1158/0008-5472.CAN-25-1076)
Supplement: Supplementary file 2 [file can-25-1076_figure_s2_suppsf2.pdf]

Figure S2. SET1B is required for sustained HIF-2 activity related to Figure 2

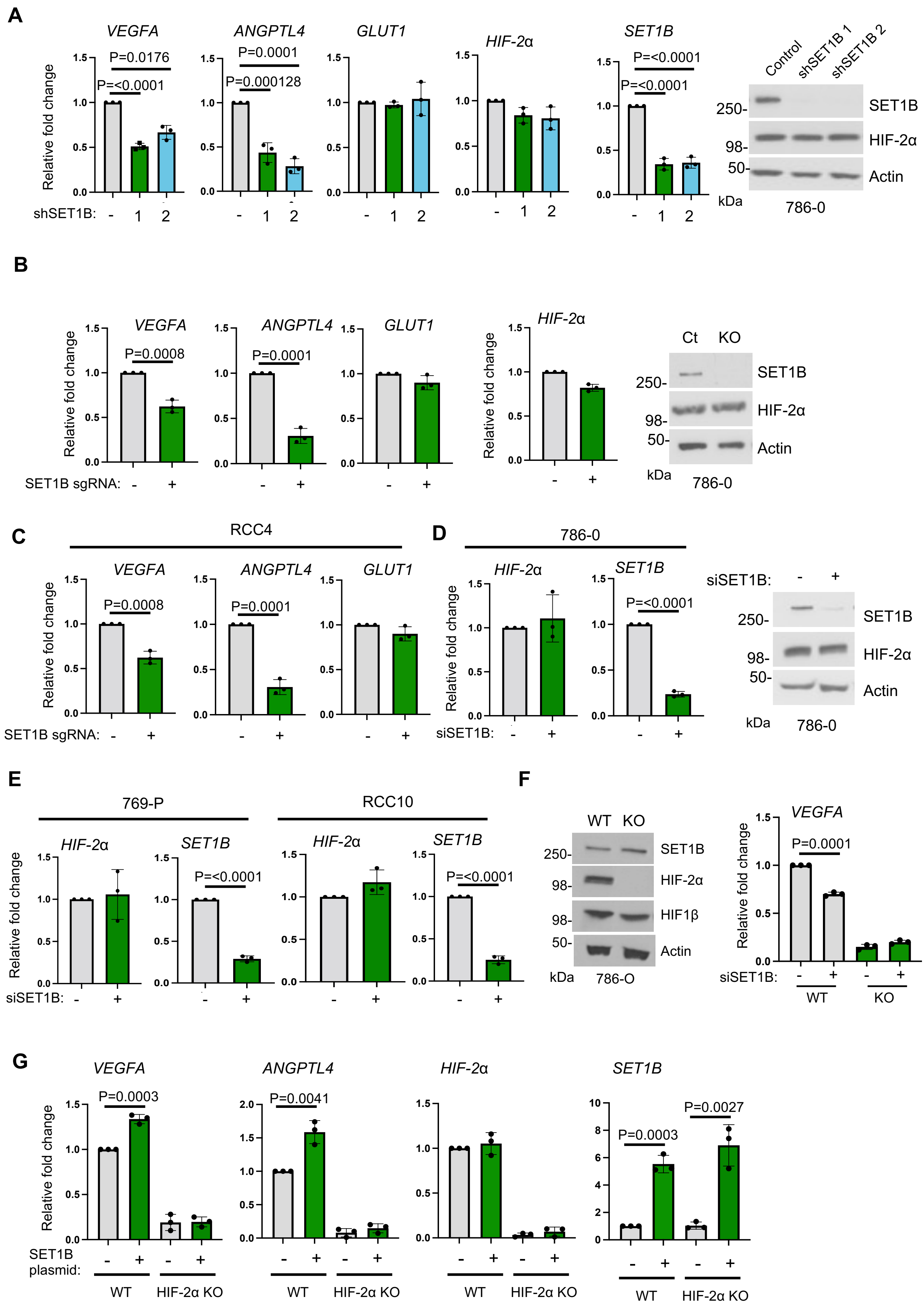

Figure S2. SET1B is required for sustained HIF-2 activity related to Figure 2 continued

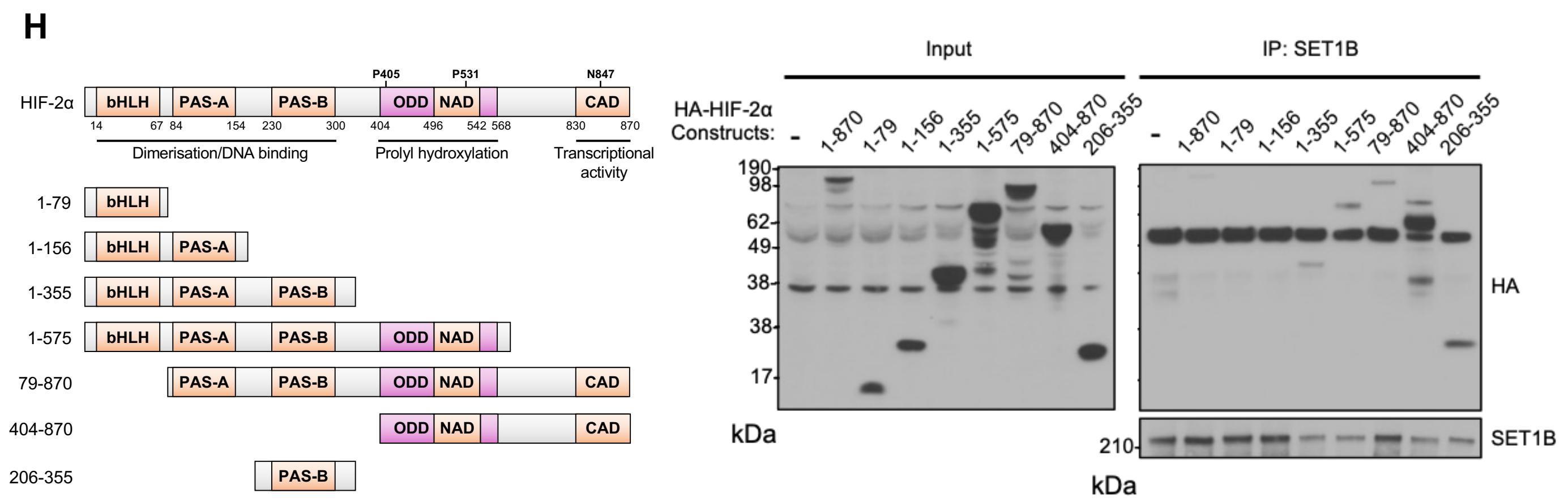

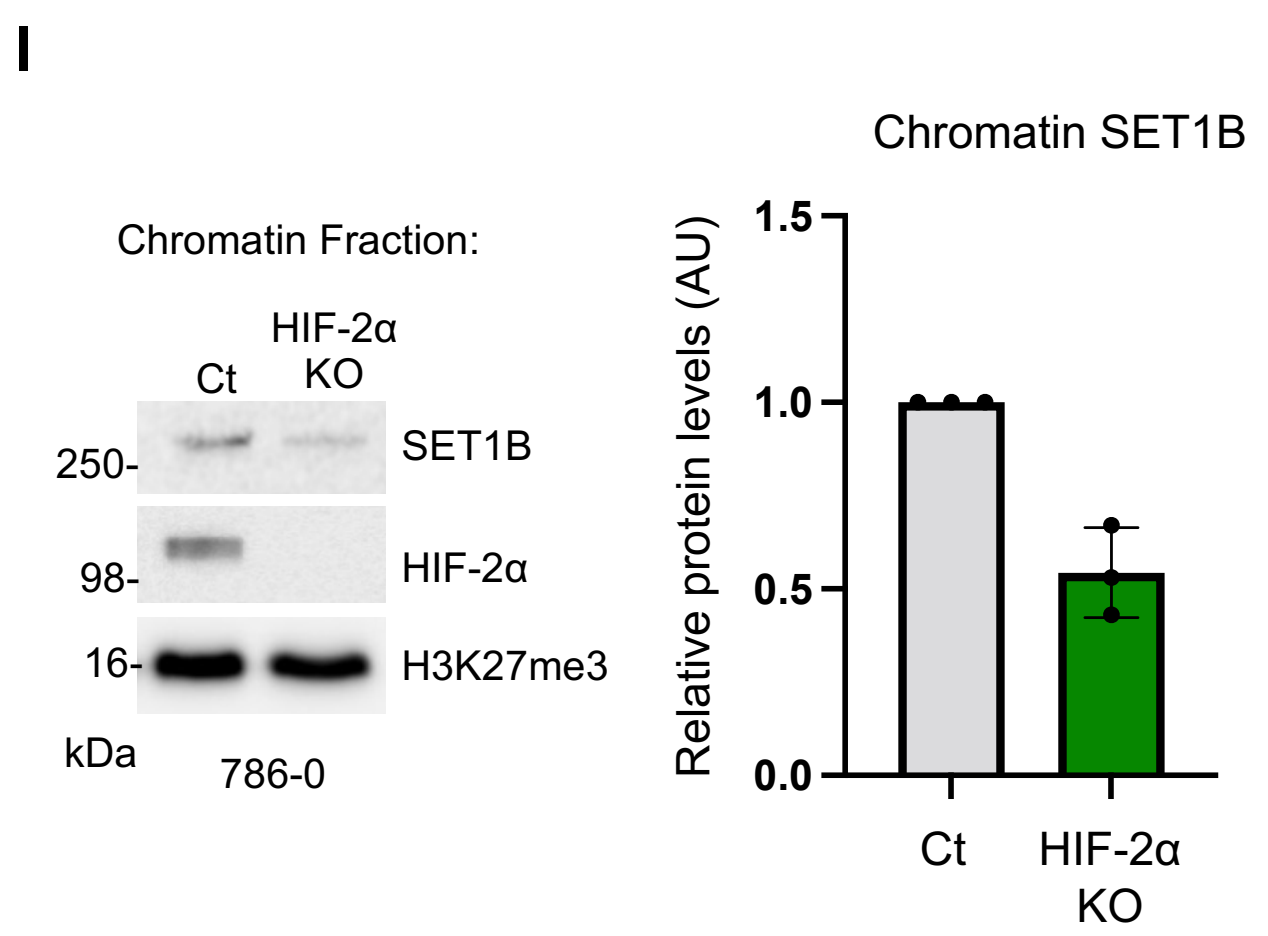

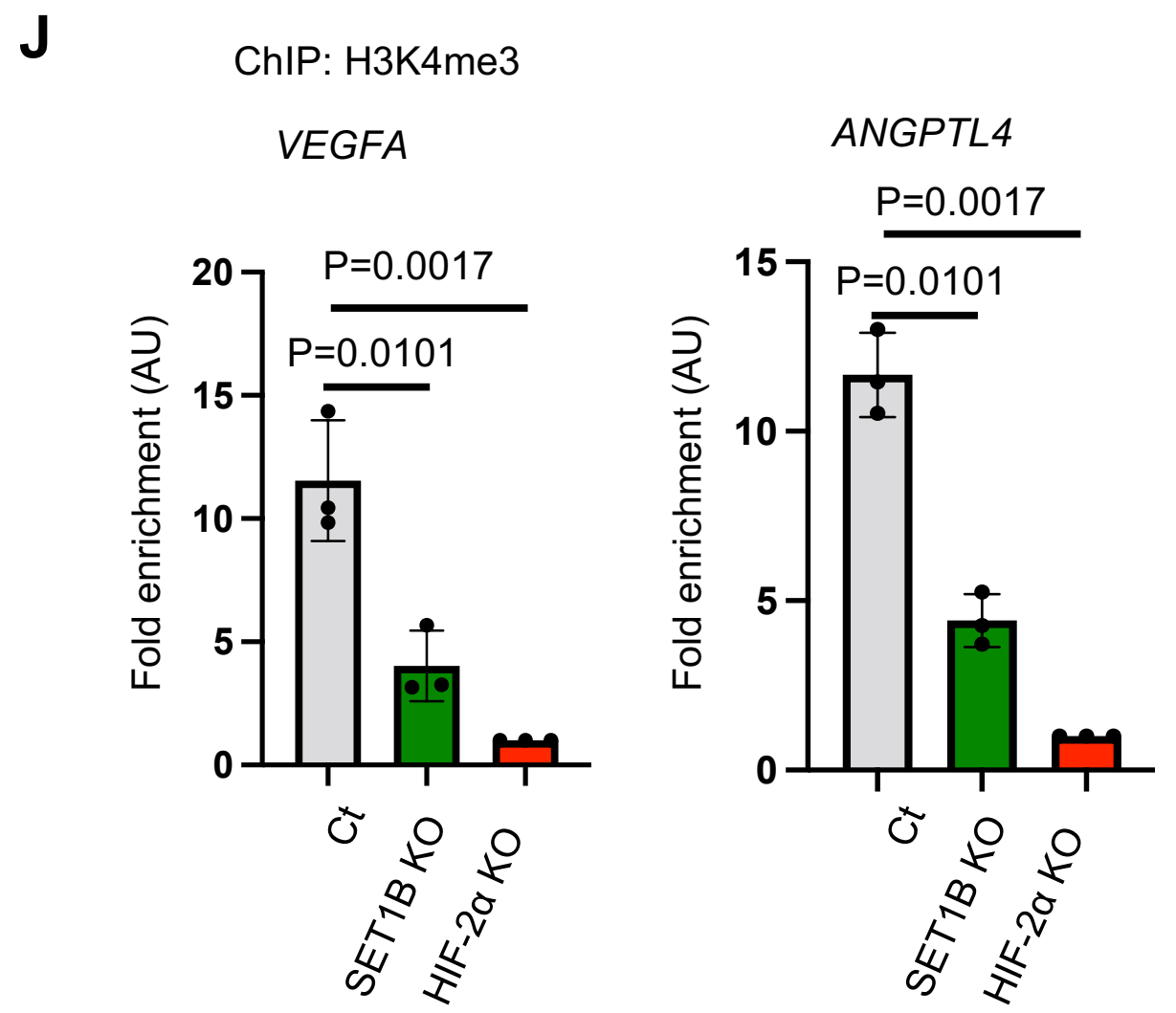

Figure S2. SET1B is required for sustained HIF-2 activity.

786-0 cells were transduced with two shRNA's targeting SET1B **(A)** or an sgRNA targeting SET1B **(B)** and the expression of HIF-2α target genes (*VEGFA, ANGPTL4, GLUT1*) and *SET1B* were assessed using qPCR. Graphs are representative for 3 biological replicates and depict the mean ± SD. Two-way ANOVA. Immunoblot conforms loss of SET1B protein following shRNA or CRISPR-mediated depletion. **(D)** RCC4 cells were transfected with a control or a SET1B targeting siRNA for 48hrs. qPCR was performed on HIF-2 target genes (*VEGFA, ANGPTL4, GLUT1*) and *SET1B* were assessed using qPCR. Graphs are representative for 3 biological replicates and depict the mean ± SD. Two-way ANOVA. **(E)** 786-0, **(F)** 769-P and RCC10 cells were transfected with a SET1B siRNA and immunoblot and qPCR was performed to assess if SET1B loss alters HIF-2α RNA or protein expression. **(G)** Immunoblot and qPCR of wildtype and HIF-2α KO 786-0 cells with indicated antibodies and qPCR was performed on *VEGFA*. Graphs are representative for 3 biological replicates and depict the mean ± SD. Two-way ANOVA. **(I)** Schematic diagram of full-length HIF-2α and truncation mutants. HEK293T cells were transfected with full length HIF-2α and the various truncation mutants using calcium phosphate for 48hrs. Cells were incubated for 6 hrs at 1% oxygen and endogenous SET1B was immunoprecipitated to assess their interaction with SET1B using immunoblotting. Immunoblot is representative of three biological replicates. Chromatin fraction was performed on wildtype and HIF-2α KO 786-0 cells before samples were immunoblotted using indicated antibodies. The ratio of SET1B and HIF-2α in the chromatin fraction was quantified using ImageJ ($n = 3$ biologically independent experiments, mean ± SD). **(K)** ChIP-PCR of H3K4me3 in wildtype, SET1B KO and HIF-2α KO 786-0 cells. ChIP-PCR was performed using primers targeting the promoters of *VEGFA* and *ANGPTL4.* Graphs are representative for 3 biological replicates and depict the mean ± SD. Two-way ANOVA.
