# Supplementary material for: SET1B Drives Sustained HIF Activity and Disease Progression in Clear-Cell Renal Cell Carcinoma
Source: Cancer Res. 2026 Apr 6;86(15):3704–19. doi: 10.1158/0008-5472.CAN-25-1076 (PMC13434302; doi:10.1158/0008-5472.CAN-25-1076)
Supplement: Supplementary file 3 [file can-25-1076_figure_s3_suppsf3.pdf]

Figure S3: SET1B regulates ccRCC progression, related to Figure 3

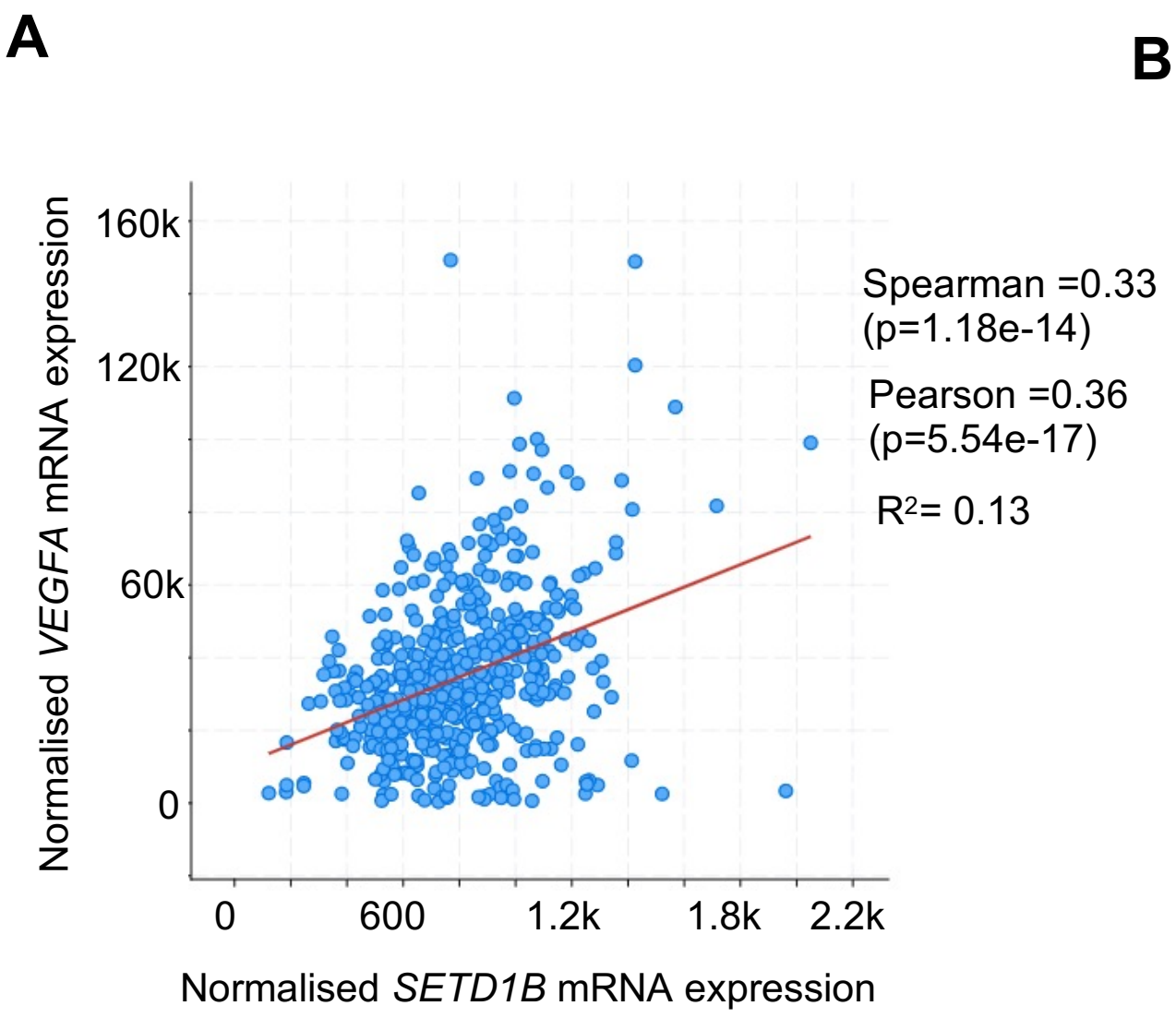

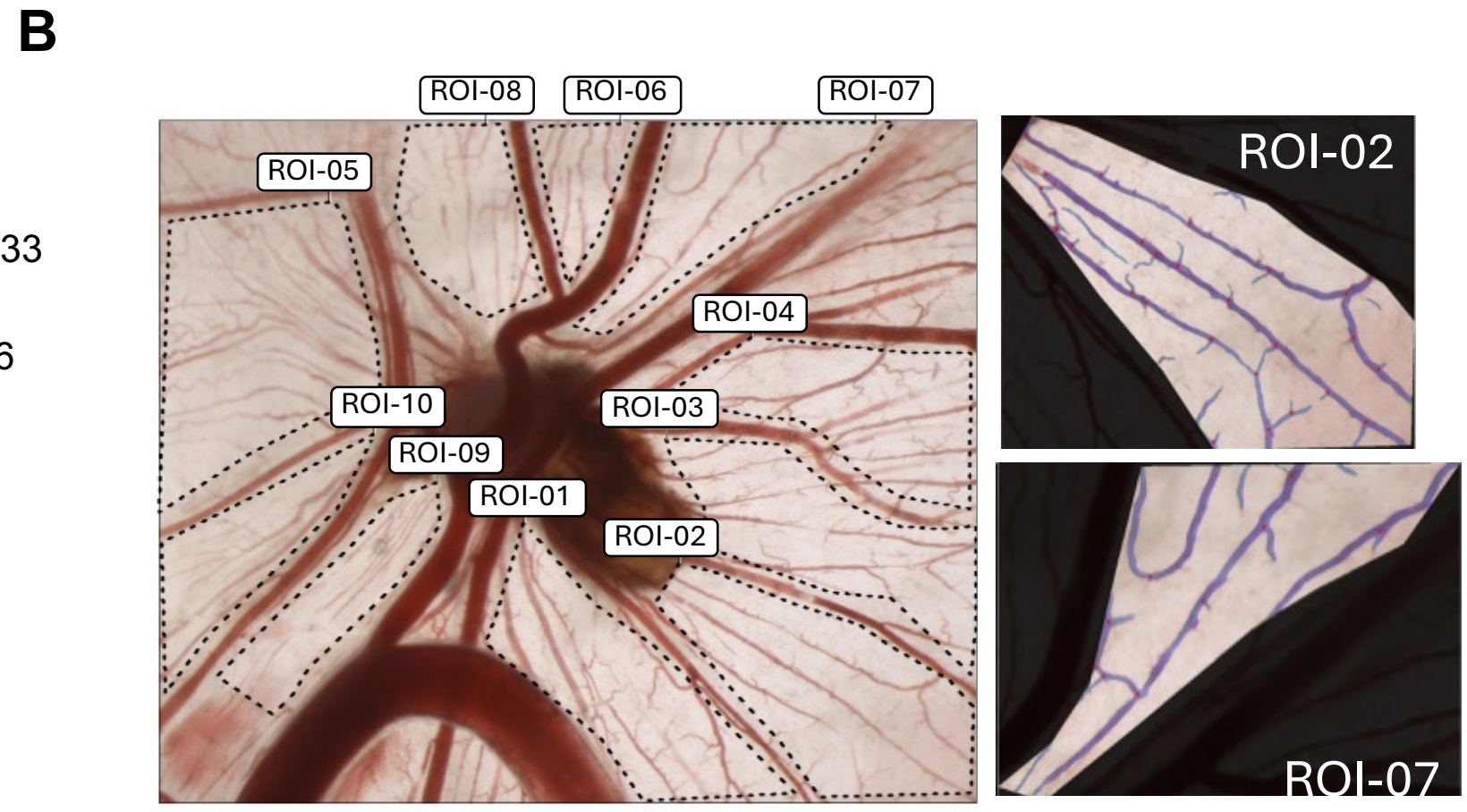

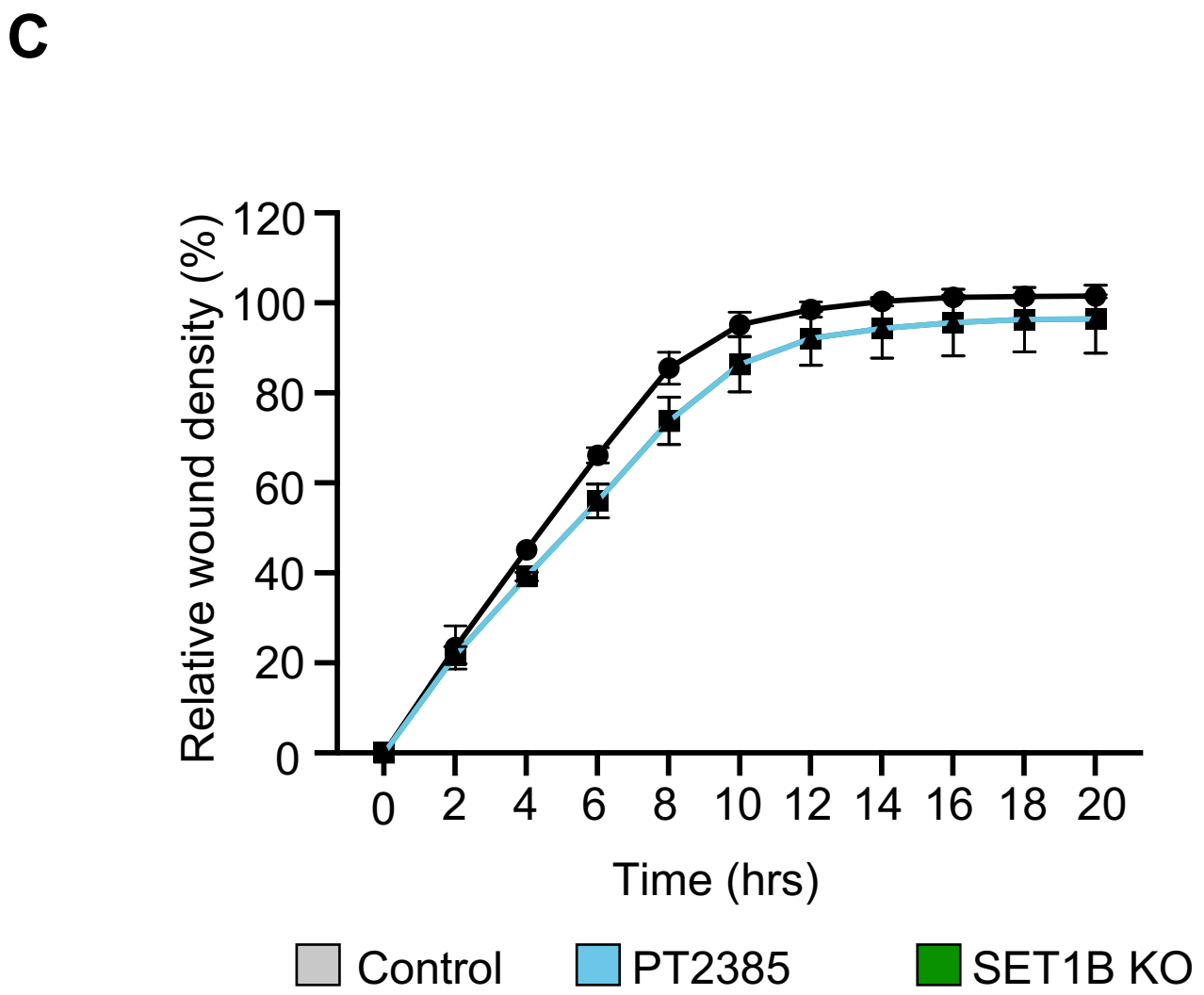

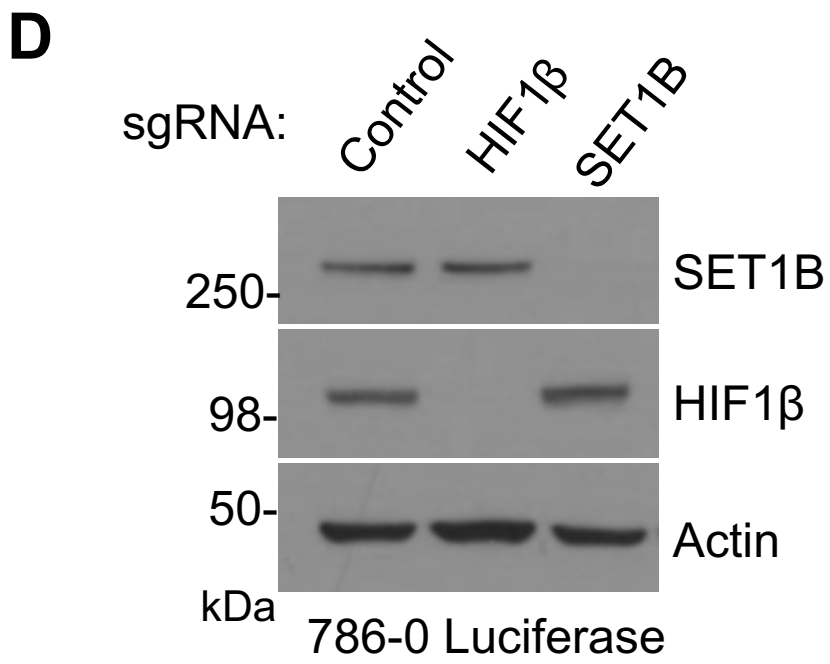

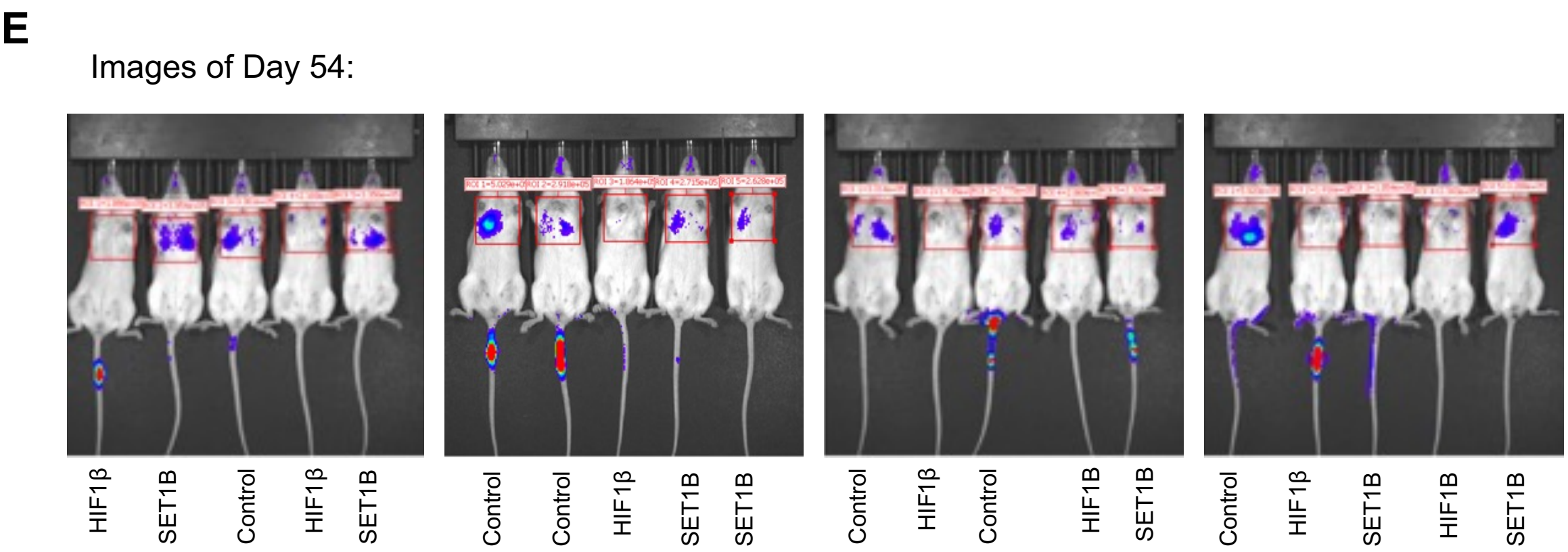

Figure S3. The effect of SET1B depletion on *VEGF* expression and on cell migration in ccRCC.

(A) Comparison of the expression of *VEGFA* mRNA vs SETD1B in ccRCC using cBioportal. **(B)** Regions of interest (ROIs) in between large blood vessels (top) were analysed using the IKOSA CAM Assay software (bottom; purple = vessels, red = branching points). Representative image corresponds to HIF1β KO in Figure 3D. **(C)** Cell migration assay**.** Control SET1B KO and PT2385 treated cells were plated at high density before a scratch was made in the well. Cell migration was measured using the Incucyte at indicated time point. Graph is representative of 3 biological replicates. **(D)** Immunoblot of control sgRNA depleted HIF1β and SET1B 786-0 luciferase cells. Immunoblot was conducted before implantation into mice. **(E)** Representative images of luciferase metastases lung metastasis in mice at day 54.
