# Supplementary material for: SET1B Drives Sustained HIF Activity and Disease Progression in Clear-Cell Renal Cell Carcinoma
Source: Cancer Res. 2026 Apr 6;86(15):3704–19. doi: 10.1158/0008-5472.CAN-25-1076 (PMC13434302; doi:10.1158/0008-5472.CAN-25-1076)
Supplement: Supplementary file 4 [file can-25-1076_figure_s4_suppsf4.pdf]

Figure S4: Loss of SET1B enhances the efficacy of HIF-2 inhibition, related to Figure 4.

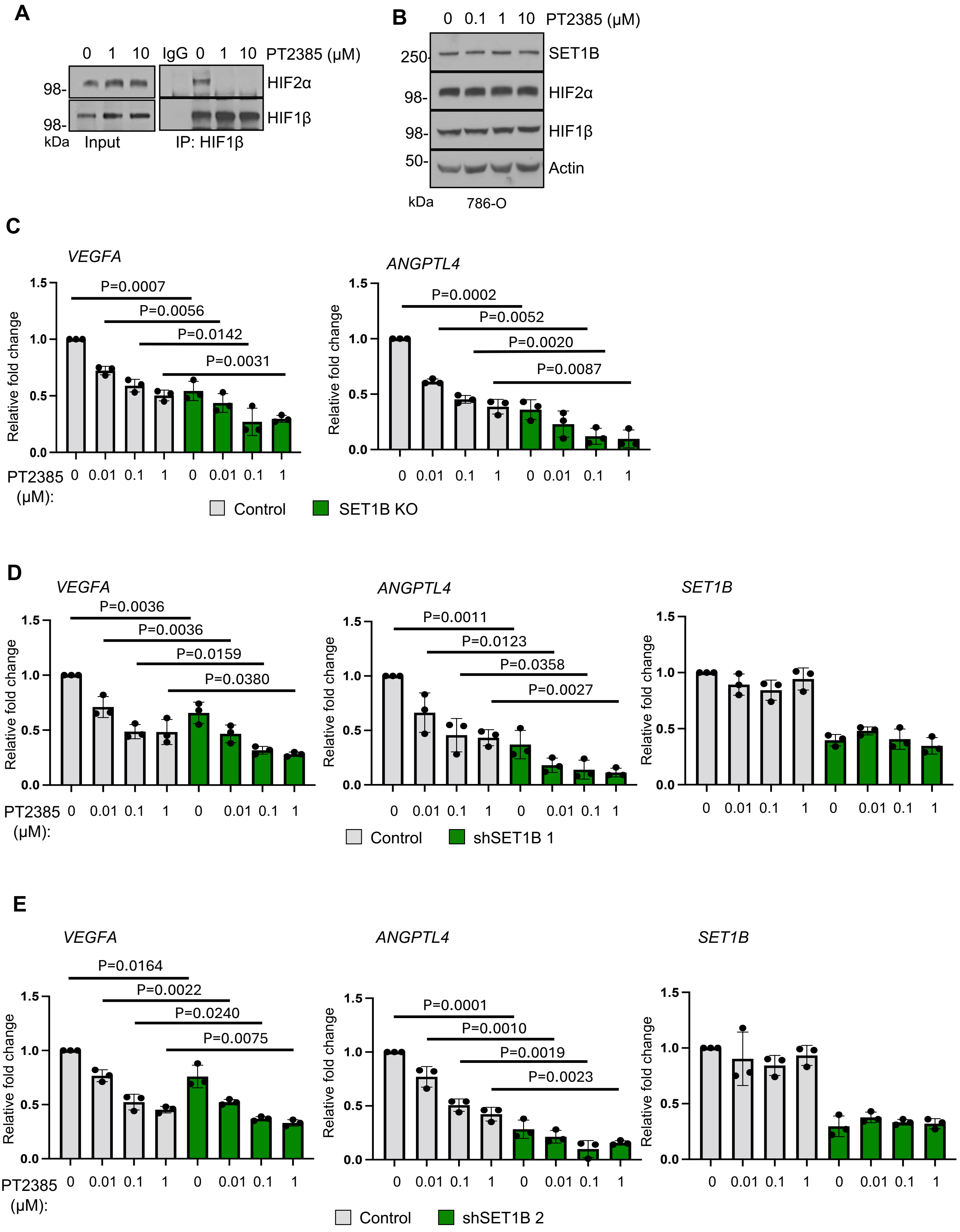

Figure S4: Loss of SET1B enhances the efficacy of HIF-2 inhibition, related to Figure 4 continued.

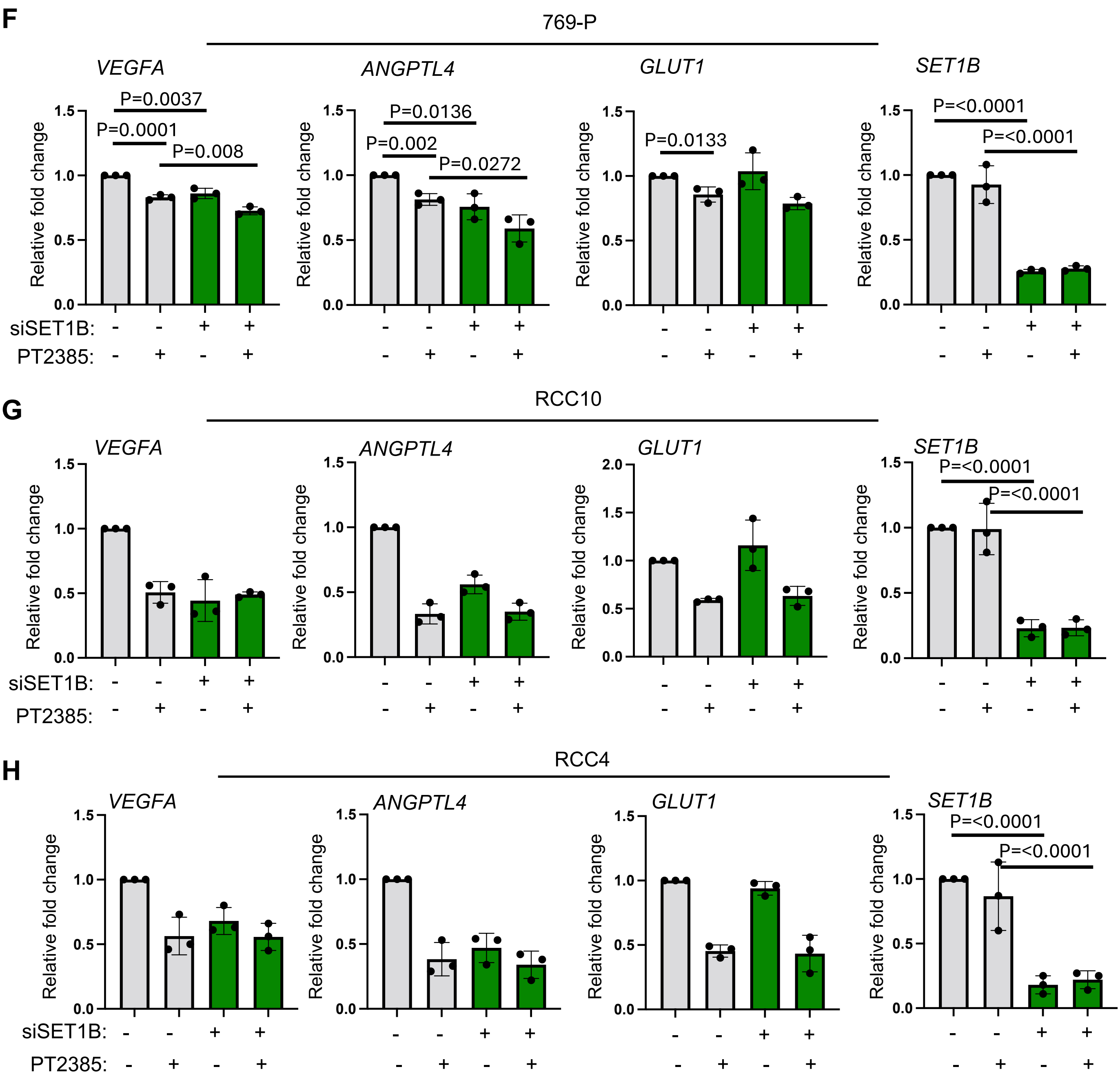

Figure S4. Loss of SET1B enhances the efficacy of HIF-2 Inhibition.

(A) HIF1β was immunoprecipitated in 786-0 cells and its interaction with HIF-2α was assessed using immunoblotting following treatment indicated concentration of PT2385 for 24 hours. Immunoblot representative of three biological replicates. **(B)** Effect of PT2385 on expression of SET1B and HIF-2α, 786-0 cells were treated with indicated concentrations of PT2385 for 24 hrs. Immunoblot is representative of three biological replicates. Wildtype 786-0 and 786-0 cells expressing an sgRNA **(C)** or two different shRNAs targeting SET1B **(D and E)** were treated with indicated concentrations of PT2385 prior to harvesting. HIF-2α activity was determined by performing qPCR of VEGFA and ANGPTL4 (n = 3 biologically independent experiments, mean ± SD). SET1B mRNA levels was analysed to validate successful depletion. **(F)** Wildtype 769-P, **(G)** RCC10 and **(H)** RCC4 cells were transfected with a control or a SET1B siRNA for 48 hrs. 24 hrs prior to harvesting cells were treated with indicated concentrations of PT2385. HIF-2α activity was determined by performing qPCR of VEGFA and ANGPTL4 (n = 3 biologically independent experiments, mean ± SD)
