# Supplementary material for: SET1B Drives Sustained HIF Activity and Disease Progression in Clear-Cell Renal Cell Carcinoma
Source: Cancer Res. 2026 Apr 6;86(15):3704–19. doi: 10.1158/0008-5472.CAN-25-1076 (PMC13434302; doi:10.1158/0008-5472.CAN-25-1076)
Supplement: Supplementary file 5 [file can-25-1076_table_s1_suppst1.docx]

**Table S1: HIF-2 Cloning Sequences**

| **HIF-2 G323E Mutant Cloning Primers** | |
| --- | --- |
| HIF2A Gibson Forward Primer | CAGTCCTCCGACAGACTGAGTCGCCCGGGGGGGATCCGCCACCATGACAGCTGAACAAGGAGAAGAAAA |
| HIF-2 Gibson Reverse Primer | CTTGCATGCCTGCAGGTCGACTCTAGAGTCGCGGCCGCTCAGGTGGCCTGGTCCAGGGCTCTG |
| HIF2A Mutant Primer Forward | ATGGGGGCTACGTGTGGCTGGAGACCCAGGAGACGGTCATCTACAACCCTCGCAACCTGC |
| HIF2A Mutant Primer Reverse | GCAGGTTGCGAGGGTTGTAGATGACCGTCTCCTGGGTCTCCAGCCACACGTAGCCCCC |

| **HIF2 Truncation mutant cloning primers** | |
| --- | --- |
| HIF2_Fw_1 | GCTTATCCTTACGACGTGCCTGACTACGCCGGATCCACAGCTGACAAGGAGAAGAAAAGGAGT |
| HIF2_Fw_79 | GCTTATCCTTACGACGTGCCTGACTACGCCGGATCCTCCGAAGCCGAAGCTGACCAGCAGATG |
| HIF2_Fw_206 | GCTTATCCTTACGACGTGCCTGACTACGCCGGATCCCCTCCTCACAATAGTCTGTGTGGCTAC |
| HIF2_Fw_404 | GCTTATCCTTACGACGTGCCTGACTACGCCGGATCCGCTCCCACCCCAGGAGACGCCATCATC |
| HIF2_Rv_870 | CTTGCATGCCTGCAGGTCGACTCTAGAGTCGCGGCCGCTCAGGTGGCCTGGTCCAGGGCTCTGAGGAG |
| HIF2_Rv_79 | CTTGCATGCCTGCAGGTCGACTCTAGAGTCGCGGCCGCTCAGGACTCGTTTTCAGAGCAAACTGAGGA |
| HIF2_Rv_156 | CTTGCATGCCTGCAGGTCGACTCTAGAGTCGCGGCCGCTCACCCAAAACCAGAGCCATTTTTGAGACT |
| HIF2_Rv_355 | CTTGCATGCCTGCAGGTCGACTCTAGAGTCGCGGCCGCTCAGGAGAACACCACGTCATTCTTCTCAAT |
| HIF2_Rv_575 | CTTGCATGCCTGCAGGTCGACTCTAGAGTCGCGGCCGCTCAGGCCAGTGGCTGGAAGATGTTTGTCAT |
