# Supplementary material for: SET1B Drives Sustained HIF Activity and Disease Progression in Clear-Cell Renal Cell Carcinoma
Source: Cancer Res. 2026 Apr 6;86(15):3704–19. doi: 10.1158/0008-5472.CAN-25-1076 (PMC13434302; doi:10.1158/0008-5472.CAN-25-1076)
Supplement: Supplementary file 6 [file can-25-1076_table_s2_suppst2.docx]

**Table S2: CRISPR sgRNAs and shRNA Sequences:**

| **CRISPR sgRNA oligonucleotide sequences:** | |
| --- | --- |
| HIF1β sgRNA | CAGTCCTCCGTCTCCTCACC |
| HIF-2α sgRNA | GCTGATTGCCAGTCGCATGA |
| SET1B sgRNA | GATGTCCAGCAACCGCCC |
| **shRNA Sequences:** | |
| shSET1B 1 | GCCGCCACGAACATCATTATG |
| shSET1B 2 | GCTTGTAGAGACGGCTGATTC |
