# Supplementary material for: SET1B Drives Sustained HIF Activity and Disease Progression in Clear-Cell Renal Cell Carcinoma
Source: Cancer Res. 2026 Apr 6;86(15):3704–19. doi: 10.1158/0008-5472.CAN-25-1076 (PMC13434302; doi:10.1158/0008-5472.CAN-25-1076)
Supplement: Supplementary file 7 [file can-25-1076_table_s3_suppst3.docx]

**Table S3: Reagents and Antibodies:**

| **Reagents and Antibodies** | **Source** | **Cat Number:** |
| --- | --- | --- |
| Mouse monoclonal anti-β actin Immunoblot: 1:20,000 | Sigma | #A2228; |
| Rabbit monoclonal anti-CFP1 Immunoblot: 1:1000 | Cell Signalling | #40672 |
| Rabbit monoclonal anti-H3K4me3 Chromatin immunoprecipitation: 1:50 | Cell Signalling | #9751 |
| Rabbit monoclonal anti-H3K36me3 Immunoblot: 1:1000 | Cell Signalling | #4909 |
| Mouse monoclonal anti-HIF-1⍺ Immunoblot: 1:1000 | BD Biosciences | #610959 |
| Rabbit monoclonal anti-HIF-2α Immunoblot: 1:1000 Chromatin immunoprecipitation: 1:50 | Cell Signalling | #59973 |
| Rabbit monoclonal anti-HIF1β Immunoblot: 1:1000 | Cell Signalling | #5537 |
| Rabbit monoclonal anti-RBBP5 Immunoblot: 1:1000 | Cell Signalling | #13171 |
| Rabbit monoclonal anti-Rpb1 Immunoblot: 1:1000 | Cell Signalling | #2629 |
| Rabbit monoclonal anti-SET1B Immunoblot: 1:1000  Immunohistochemistry: 1:100 | Cell Signalling | #20055 |
| Rabbit monoclonal anti-WDR82 Immunoblot: 1:1000 | Cell Signalling | #99715 |
| Peroxidase-AffiniPure Goat Anti-Mouse IgG Immunoblot secondary: 1:20,000 | Jackson | 115-035146 |
| Peroxidase-AffiniPure Goat Anti-Rabbit IgG Immunoblot secondary: 1:20,000 | Jackson | 111-035045 |

| **Reagent** | **Source** | **Cat Number:** |
| --- | --- | --- |
| PT2385 | Caymen Chemicals | CAY21325-5 mg |
