# Supplementary material for: SET1B Drives Sustained HIF Activity and Disease Progression in Clear-Cell Renal Cell Carcinoma
Source: Cancer Res. 2026 Apr 6;86(15):3704–19. doi: 10.1158/0008-5472.CAN-25-1076 (PMC13434302; doi:10.1158/0008-5472.CAN-25-1076)
Supplement: Supplementary file 8 [file can-25-1076_table_s4_suppst4.docx]

**Table S4: qPCR and ChIP primers:**

| **qPCR Primers:** | |
| --- | --- |
| Actin Forward | CTGGGAGTGGGTGGAGGC |
| Actin Reverse | TCAACTGGTCTCAAGTCAGTG |
| AKAP12 Forward | GAGATGGCTACTAAGTCAGCGG |
| AKAP12 Reverse | CAGTGGGTTGTGTTAGCTCTTC |
| ANGPTL4 Forward | TCCGTACCCTTCTCCACTTG |
| ANGPTL4 Reverse | AGTACTGGCCGTTGAGGTTG |
| CA9 Forward | GCCGCCTTTCTGGAGGA |
| CA9 Reverse | TCTTCCAAGCGAGACAGCAA |
| GLUT1 Forward | CCAGGGTAGCTGCTGGAGC |
| GLUT1 Reverse | TGGCATGGCGGGTTGT |
| MAFF Forward | CCAGAAGGCAGAGGGTTGTAGTGA |
| MAFF Reverse | AGGAGCCGGGCAATATTTTTTA |
| SET1A Forward | CAGTGGCGGAACTACAAGCTC |
| SET1A Reverse | CATAGCGGTACACCTTCTGAGA |
| SET1B Forward | AGGGGCATCATAAACTGTACCG |
| SET1B Reverse | GGGGATCTTCGACAATTTCCAC |
| VEGFA Forward | TACCTCCACCATGCCAAGTG |
| VEGFA Reverse | ATGATTCTGCCCTCCTCCTTC |
| **ChIP qPCR Primers:** | |
| ANGPTL4 Forward | ATTTGCTGTCCTGGCATC |
| ANGPTL4 Reverse | CCAGCTCATTCTCTGGAATC |
| CA9 Forward | GACAAACCTGTGAGACTTTGGCTCC |
| CA9 Reverse | AGTGACAGCAGCAGTTGCACAGTG |
| CA9 Exon 3 Forward | CCCGCCCTGGCCCCGGGTGT |
| CA9 Exon 3 Reverse | GGGGCGCAGGGCCGGGCAGA |
| VEGFA Forward | CCTTTGGGTTTTGCCAGA |
| VEGFA Reverse | CCAAGTTTGTGGAGCTGA |
| VEGFA Exon 3 Forward | CATCCAACAGCCAGGGGGAC |
| VEGFA Exon 3 Reverse | CAGCCCCCGCATCGCATCAG |
